# Sishen Pill and its active phytochemicals in treating inflammatory bowel disease and colon cancer: an overview

**DOI:** 10.3389/fphar.2024.1375585

**Published:** 2024-04-08

**Authors:** Boxun Zhang, Yingying Cheng, Qin Jian, Sirui Xiang, Qi Xu, Chuchu Wang, Chuan Yang, Junzhi Lin, Chuan Zheng

**Affiliations:** ^1^ Department of Endocrinology, Hospital of Chengdu University of Traditional Chinese Medicine, Chengdu, China; ^2^ State Key Laboratory of Southwestern Chinese Medicine Resources, College of Pharmacy, Chengdu University of Traditional Chinese Medicine, Chengdu, China; ^3^ TCM Regulating Metabolic Diseases Key Laboratory of Sichuan Province, Hospital of Chengdu University of Traditional Chinese Medicine, Chengdu, China; ^4^ College of Basic Medicine, Chengdu University of Traditional Chinese Medicine, Chengdu, China; ^5^ Department of Dermatology, Hospital of Chengdu University of Traditional Chinese Medicine, Chengdu, China; ^6^ Sichuan Provincial Engineering Research Center of Innovative Re-development of Famous Classical Formulas, Tianfu TCM Innovation Harbour, Chengdu University of Traditional Chinese Medicine, Chengdu, China

**Keywords:** inflammatory bowel disease, colon cancer, Sishen Pill, molecular mechanism, natural product

## Abstract

The incidence of inflammatory bowel disease (IBD) and the associated risk of colon cancer are increasing globally. Traditional Chinese medicine (TCM) treatment has unique advantages. The Sishen Pill, a common Chinese patented drug used to treat abdominal pain and diarrhea, consists mainly of Psoraleae Fructus, Myristicae Semen, Euodiae Fructus, and Schisandra Chinensis. Modern research has confirmed that Sishen Pill and its active secondary metabolites, such as psoralen, myristicin, evodiamine, and schisandrin, can improve intestinal inflammation and exert antitumor pharmacological effects. Common mechanisms in treating IBD and colon cancer mainly include regulating inflammation-related signaling pathways such as nuclear factor-kappa B, mitogen-activated protein kinase, phosphatidylinositol 3-kinase, NOD-like receptor heat protein domain-related protein 3, and wingless-type MMTV integration site family; NF-E2-related factor 2 and hypoxia-inducible factor 1α to inhibit oxidative stress; mitochondrial autophagy and endoplasmic reticulum stress; intestinal immune cell differentiation and function through the Janus kinase/signal transducer and activator of transcription pathway; and improving the gut microbiota and intestinal barrier. Overall, existing evidence suggests the potential of the Sishen pill to improve IBD and suppress inflammation-to-cancer transformation. However, large-scale randomized controlled clinical studies and research on the safety of these clinical applications are urgently required.

## 1 Introduction

Inflammatory bowel disease (IBD) is a chronic disease of the intestine that mainly includes ulcerative colitis (UC) and Crohn’s disease (CD). Its incidence has shown an upward trend worldwide (Ng et al., 2017). UC, the most important type of IBD, progresses gradually from the rectum to the proximal segments of the colon, with its lesions often localized to the mucosal epithelium. UC can occur in any part of the gastrointestinal tract and is commonly found in the terminal ileum and right colon. In addition to common discomfort symptoms, such as abdominal pain, diarrhea, and bloody stools, IBD is associated with an increased risk of various metabolic diseases, such as diabetes (Lu et al., 2020; Li et al., 2021b), acute coronary syndrome (D'Ascenzo et al., 2023; Zaka et al., 2023), nonalcoholic fatty liver disease (Chen et al., 2020c), and autoimmune skin diseases (Fu et al., 2018) such as rheumatoid arthritis and psoriasis. More importantly, IBD can increase the risk of various cancers, such as colon cancer (Gatenby et al., 2021; Piovani et al., 2022). A survey of patients with UC revealed that the estimated cumulative risk of UC-associated colorectal cancer was 0.7% within 10 years, but by 30 years, the risk rose to 33.2% (Kim et al., 2009). Treatment of IBD with 5-aminosalicylates can significantly reduce the incidence of colon cancer (Bonovas et al., 2017; Hsiao et al., 2022). In recent studies, targeted nutritional interventions (Cassotta et al., 2023), probiotics, and other intestinal microecological agents (Lee et al., 2022) were found to be effective in treating colitis-associated colon cancer (CACC). The process of IBD transformation into cancer involves complex molecular mechanisms, such as gene mutations, epigenetic alterations, persistent chronic inflammation, gut microbiota disorders, and others (Xue et al., 2018). Further exploration is warranted to limit intestinal inflammation and inhibit its transformation into colon tumors.

Natural botanical drugs have the therapeutic advantage of multiple pathways and multiple targets; numerous studies have confirmed that botanical drugs or their extracts could improve IBD, inhibit its progression to colon cancer, exerting an integrated pharmacological “anti-inflammatory + anti-cancer” effect (Yang et al., 2023b). Traditionally, the Sishen Pill is a Chinese patent drug commonly used to treat diarrhea and is mainly composed of Psoraleae Fructus, Myristicae Semen, Euodiae Fructus, and Schisandrae Chinensis at a dosage ratio of 4 : 2: 2 : 1. Jujubae Fructus and Zingiberis Rhizoma were also used as excipients in this formula. In traditional Chinese medicine (TCM), Sishen Pill is believed to “warm the kidneys to dispel cold and astringing the intestines to stop diarrhea.” modern clinical studies showed that it could effectively treat IBD and other intestinal inflammatory injury (Li et al., 2018; Long and Cao, 2021b; Xu et al., 2022b). The main active metabolites in this formula, such as myristicin (Ismail Abo El-Fadl and Mohamed, 2022), psoralen (Zhou, 2020), deoxyschizandrin (Zhang et al., 2016), evodiamine (Ding et al., 2020), and others could improve the intestinal mucosal damage caused by IBD through various molecular mechanisms. Recent studies also found that the Sishen Pill can effectively treat colon cancer (Jiang et al., 2023) and prevent the progression of inflammatory cancer transformation (Cao et al., 2012; Cao, 2013; Cao et al., 2013); various metabolites in this formula could also suppress the growth of colonic tumor cells. This review comprehensively summarizes the experimental research on the treatment of IBD and colon cancer with Sishen Pill and its active phytochemicals, screens for core effective phytochemicals, clarifies key targets of action, generalizes the potential common molecular mechanism of Sishen Pill to treat IBD and colon cancer, and proposes a future research outlook based on the current research.

## 2 Metabolites investigation of Sishen Pill

**FIGURE 1:**
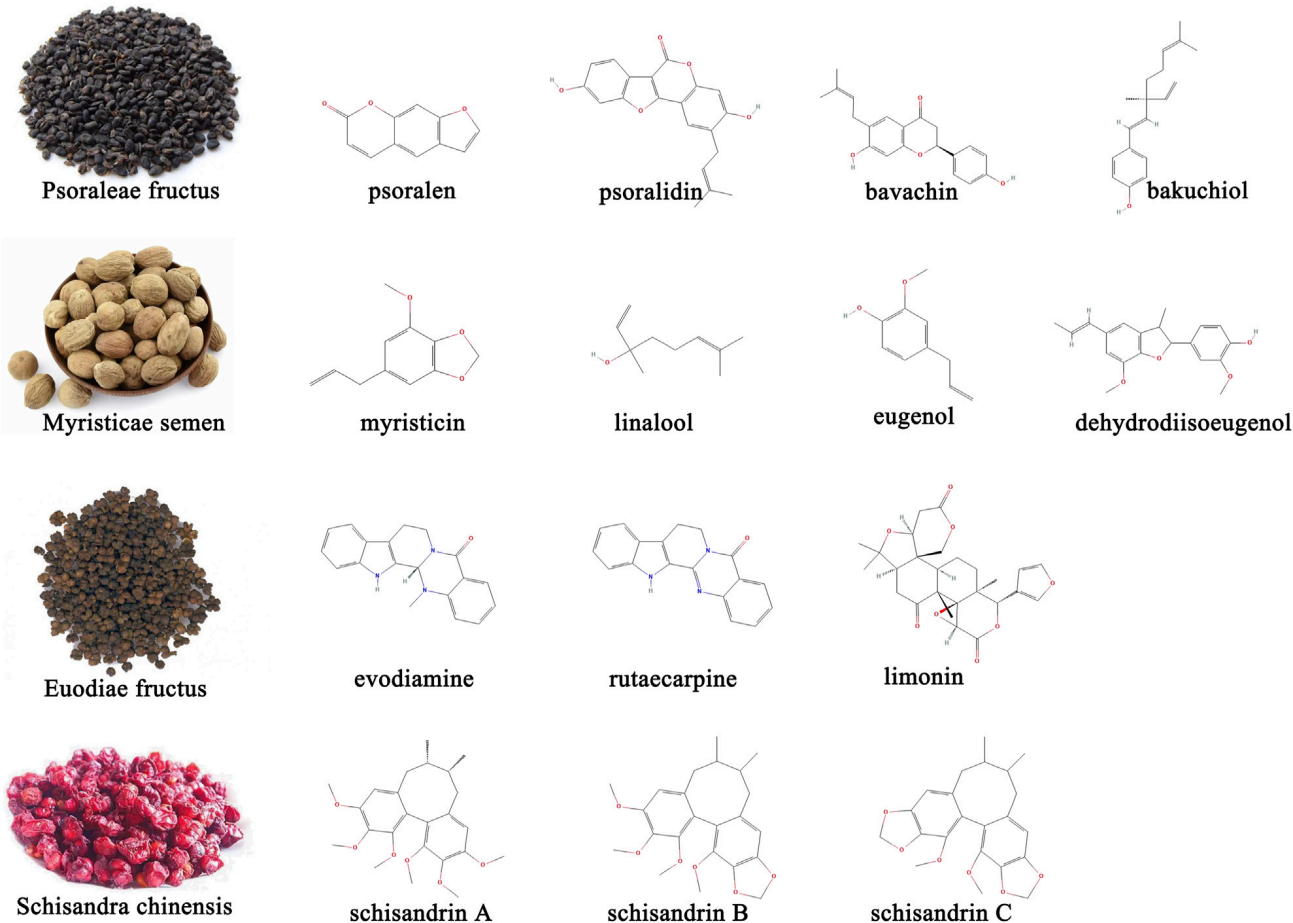
Representative active metabolites in Sishen Pill with potential therapeutic effects on IBD and/or colon cancer.

The earliest records of the Sishen Pill can be traced back to the *Hua Tuo Shen Yi Mi Zhuan* during the Han Dynasty. The main disease it treats is “predawn diarrhea” (Wang et al., 2015). Modern research has found that this formula not only treats diarrhea but also has curative effects on multiple intestinal diseases such as UC (Long and Cao, 2021a), irritable bowel syndrome (Li et al., 2018a), colorectal cancer (Sun et al., 2021), and extraintestinal diseases such as depression (Luo et al., 2023) and breast cancer (Xu et al., 2022a). The multiple active metabolites contained in the Sishen Pill determine its multi-target therapeutic effects. Several studies have applied advanced technology to analyze qualitative or quantitative the metabolites in the Sishen Pill. Briefly, high performance liquid chromatography (HPLC) (Wei et al., 2021; Guo et al., 2023), HPLC-electrospray ionization-tandem mass spectrometry (HPLC-ESI-MS/MS) (Zhang et al., 2018), and flash evaporation-gas chromatography/mass spectrometry (FE-GC/MS) (Huang et al., 2019) have been used to identify effective metabolites in this formula. Sishen Pill contains various effective metabolites such as coumarins, lignin, alkaloids, terpenoids, and others (Guo et al., 2023). The main metabolites with potential therapeutic effects on IBD and/or colon cancer are shown in Figure 1.

Psoraleae Fructus is the dried ripe fruit of *Psoralea corylifolia* Linn. The Leguminosae family and its metabolites include coumarins, flavonoids, benzofurans, monoterpenes, and some trace elements (Mu et al., 2018). Other studies focused on psoralen, isopsoralen, and psoralidin in coumarins; bavachin, bavachinin, and neobavaisoflavone in flavonoids; and bakuchiol in monoterpenoids (Chopra et al., 2013). In addition to antibacterial, anti-inflammatory, antitumor, antiviral, and antioxidant effects, Psoraleae Fructus can regulate bone cell metabolism, enhance skin pigmentation, and act like estrogen, expanding its utility in orthopedics, dermatology, and gynecology (Ren et al., 2020; Sharifi-Rad et al., 2020). The coumarin content in Psoraleae Fructus is an important indicator that the Sishen Pill meets quality standards (Huang et al., 2019). Several studies have confirmed that intestinal bacteria play an important role in metabolic processes. Wang et al. developed a rapid, sensitive, and selective ultra-performance liquid chromatography-tandem mass spectrometry (UPLC-MS/MS) method and found that psoralenoside and isopsoralenoside could be metabolized to psoralen and isopsoralen by gut microbiota through de-glucosylation (Wang et al., 2014). Furthermore, Liu et al. investigated the metabolic profiles of psoralen and isopsoralen under intestinal conditions and confirmed that some metabolites, such as 6,7-furano-hydrocoumaric acid and 5,6-furano-hydrocoumaric acid, have stronger activities in antioxidant stress and as anti-inflammatories (Liu L. et al., 2019).

Myristica Semen, the dried seeds of *Myristica fragrans* Houtt. plants in the Myristiceae family are a common TCM medicinal and edible homologous that contains lignans such as dehydrodiisoeugenol and macelignan; phenylpropanoids such as myristicin, eugenol, isoeugenol, and elemicin; and terpene alcohols such as linalool, all found to have multiple pharmacological properties (Liu et al., 2023). In addition to the therapeutic effects on the digestive system, such as peptic ulcer and diarrhea, Myristicae Semen has also been shown to be active against Parkinson’s disease and has anti-depressant, anti-epileptic, and anti-dementia effects (Liu et al., 2023). The combination of Myristicae Semen and Psoraleae Fructus, known as the traditional Ershen Pill formula, is also used to treat intestinal diseases such as diarrhea and abdominal cold pain. Gao et al. (2017) used HPLC to “fingerprint” Ershen Pill-medicated serum and found that psoralen, isopsoralen, bakuchiol, corylin, and dehydrodiisoeugenol were the main metabolites absorbed into the blood.

Euodiae Fructus is a nearly ripe, dry fruit of the Rutaceae plant *Euodia rutaecarpa* (Juss.) Benth. or *E. rutaecarpa* (Juss.) var. *officinalis* (Dode) Huang, or *E. rutaecarpa* (Juss.) Benth. var. *bodinieri* (Dode) Huang; it contains mainly alkaloids, terpenoids, flavonoids, phenylpropanoids, anthraquinone, and sterols; research has now focused on metabolites such as evodiamine, rutaecarpine, rutaevine, and limonin (Kong et al., 2023). Euodiae Fructus is widely used in clinical practice and has multiple effects, including pain relief, anti-inflammatory effects, gastrointestinal protection, antitumor effects, central nervous system protection, cardiovascular protection, and glycolipid metabolism regulation. Recently, to solve the problems of low solubility and bioavailability of evodiamine, attempts have been made to develop novel phospholipid and nanocomplex drug carriers to deliver evodiamine, achieve better clinical efficacy and reduce side effects (Luo et al., 2021).

Schisandrae Chinensis originates from the dried and ripe fruits of the Magnoliaceae plant *Schisandra Chinensis* (Turcz.) Baill, or *Schisandra Sphenanthera* Rehd. et Wils; the former is called *Schisandrae Sphenantherae Fructus,* whereas the latter is called *Schisandrae Chinensis Fructus*. The effective metabolites of Schisandrae Chinensis contain lignans, volatile oils, polysaccharides, organic acids, terpenoids, and flavonoids. Among them, lignans are considered the primary active metabolites, including mainly schizandrin A, schizandrin B, schizandrin C, schizandrol A, schizandrol B, schistenherin A, and schistenherin B. Studies found that schisandrins could regulate the central nervous, cardiovascular, digestive, endocrine, and immune systems, and are often used for sleep promotion, regulation of glucose and lipid metabolism, and as anti-inflammatory and anti-diarrhea agents (Xing et al., 2021). Similar to evodiamine, schisandrins have relatively low bioavailability; new technologies such as self-emulsifying drug delivery systems and solubility have been improved to some extent (Shao et al., 2010).

## 3 Research progress on Sishen Pill in the treatment of IBD and colon cancer

### 3.1 Sishen Pill in the treatment of IBD

The clinical efficacy of the Sishen Pill in treating UC has been confirmed by multiple clinical studies. *Long* et al. conducted a meta-analysis of nine randomized controlled trials (RCTs) including 680 patients and found that, compared to sulfasalazine and mesalazine, the combined use of Sishen Pill could effectively improve the effectiveness of treatment, reduce C-reactive protein levels, and have a lower incidence of adverse reactions (Long and Cao, 2021b), however, among the original studies included in this meta-analysis, different studies adopted different forms of administration of Sishen Pills (oral or enema), and it remains to be further explored which administration route can achieve better therapeutic effects. *Zhang* et al. used network pharmacology and bioinformatics methods to screen 22 key targets of the Sishen Pill in treating UC (Zhang et al., 2019) and suggested that it could improve intestinal inflammatory state, repair intestinal mucosal injury, and inhibit disease progression by regulating multiple targets, however, further experimental research is needed to confirm the relevant conclusions based on bioinformatics analysis. Table 1 lists the relevant basic research progress on the Sishen Pill for the treatment of IBD. Briefly, several studies focused on the inhibitory effects of Sishen Pill on the toll-like receptor (TLR): *Huang* (Huang et al., 2021) and *Zhao* (Zhaohua et al., 2022) found that the formula could inhibit expression levels of myeloid differentiation factor 88 (MyD88), interleukin-1 receptor associated kinase 4 (Irak4), and nuclear factor-kappa B(NF-κB) by down-regulating the activation of TLR2. *Wang* (Wang et al., 2019a) and *Ge* (Ge et al., 2022) confirmed that TLR4 was the key target, and downregulating TLR4 could inhibit the occurrence of subsequent inflammatory responses through MyD88-dependent and MyD88-independent pathways. In addition, *Zhang* (Zhang et al., 2021c), *Wang* (Wang et al., 2022a) and *Zhao* (Zhao et al., 2013) explored the molecular mechanism of the Sishen Pill in inhibiting the inflammatory response and promoting intestinal mucosal repair via phosphatidylinositol 3-kinase (PI3K)/protein kinase B (PKB/Akt), Janus kinase (JAK)/signal transducer and activator of transcription 5 (STAT5), and mitogen activated protein kinase (MAPK) signal pathways. Moreover, the regulation of intestinal immune cells by Sishen Pill mainly manifests in different subsets of T lymphocytes and regulatory T cells (Treg) (Liu et al., 2016), helper T cells (Th) (Liu et al., 2016), follicular helper T cells (Tfh) (Liu et al., 2020), follicular regulatory T cells (Tfr) (Huang et al., 2022; Kang et al., 2022), memory T cells (TM) (Ge et al., 2020), and dendritic cells (Liu et al., 2022). For regulating the gut microbiota, Sishen Pill has been shown to increase the relative abundance of beneficial bacteria, such as *Lactobacillus* and *Akkermansia*, and to promote an increase in intestinal butyrate content (Chen et al., 2020b; Wang et al., 2022b; Ge et al., 2022). In summary, the above studies have elucidated the mechanism of action of Sishen Pill in treating IBD from different perspectives, but there is still a lack of deeper exploration on the key targets of action, and the application of molecular inhibitors/activators or gene knockout animal model and other experimental methods is necessary and anticipated in future research.

**TABLE 1:** Pharmacological effects and molecular mechanisms of Si Shen Wan in the treatment of IBD.

Experimental model	Dosage form	Dosage	Pharmacological effect	Molecular mechanism	References
BALB/c mice	formula granules	2.5 g/kg/d	Regulating immune cells: Treg cell↑, Tfr cell↑, PD-1 and PD-L1 cells↓, Tfh9 and Tfh17 cells↓	Inhibiting STAT/SOCS signaling pathway: protein expression levels of p-STAT3, STAT3, p-STAT6 and STAT6 are decreased, and protein expression level of SOCS are increased	Huang et al. (2022), Kang et al. (2022)
BALB/c mice	volatile oil	0.075 g/kg/d	Regulating inflammatory factors: IL-10↑, IL-4↓, IL-17A↓, IL-21↓, IFN-γ↓	Inhibiting TLR/MyD88 signaling pathway: the levels of TLR2, MyD88, Rac1, IRAK4, IRAK1, TRAF6, TAB1, TAB2, MKK6, p38MAPK, and CREB proteins are downregulated	Huang et al. (2021)
Wistar rat	water decoction	2.5 g,5 g, 10 g/kg/d	Regulating oxidative stress and immune factors: lgE↓, MDA↓, IL-2↑, SOD↑, FT3↑, FT4↑	Regulating TLR4/IRAK-M signaling pathway: the protein expression level of TLR-4 is downregulated and IRAK-M protein is upregulated	Wang et al. (2019a)
Wistar rat	water pill	0.8 g,1.6 g and 3.2 g/kg/d	Regulating inflammatory factors: IL-1β↓, IL-10↑	Inhibiting PI3K/Akt/mTOR signaling pathway: the levels of p-PI3K, p-Akt, p-mTOR are decreased	Liu et al. (2021)
BALB/c mice	water pill	5 g/kg/d	Regulating the dendritic cell immunity: CD40↓, CD24↓, CD135↓, CD107b↓, CD115↓, CCR6↓, CD172a↑, F4/80↑	Inhibiting PI3K/Akt/mTOR signaling pathway: the level of PI3K, Akt, p-Akt, mTOR, p-mTOR, Raptor and Rictor are decreased	Liu et al. (2022)
SD rat	water pill	2.5 g/kg/d	Regulating T lymphocyte subsets: CD4^+^T cell↓, CD8^+^T cell↑, CD4/CD8↓, CD4^+^CD25^+^T cell↑, CD4^+^CD25^+^Foxp3^+^T↑, Th17 cell↓, Treg/Th17↑	Regulating the expression of RORγt and STAT5a: the protein expression of RORγt is decreased and STAT5a is increased	Liu et al. (2016)
BALB/c mice	water pill	2.5 g/kg/d	Reduce inflammatory response: TLR-2↓, TLR-4↓	Regulating the gut microbiota disorders: the abundance of pathogenic bacteria such as *Eubacterium_fissicatena* was downregulated, and the abundance of beneficial strains for protecting the intestinal mucosa, such as *Lachnospiraceae_NK4A136*, *Muribaculaceae* and *Akkermansia* was upregulated	Jin et al. (2023)
C57BL/6	Ethanol extract	20 g/kg/d,40 g/kg/d	Regulating inflammation and oxidative stress factors: IL-6↓, TNF-α↓, MDA↓ ROS↓, T-AOC↑	Regulating the Nrf2/HO-1 signaling pathway: protein and mRNA expression levels of Nrf2, HO-1, NQO-1 upregulated	Zhang et al. (2021b)
SD rat	water decoction	6 g,12 g,24 g/kg/d	Regulating inflammation and immune factors: IL-6↓, IL-17↓, STAT3↓, IL-10↑, TGF-β1↑, PPARγ↑, the proportion of Th17 cells↓, the proportion of Treg cells↑	Regulating the gut microbiota disorders: the relative abundance of *Lactobacillus* and the concentration of butyric acid are increased	Wang et al. (2022b)
BALB/c mice	water pill	2.5 g/kg/d	Regulating inflammatory factors: CD11c^+^CD103^+^E-cadherin^+^ cells↓, IL-1β↓, IL-4↓,IL-9↓, IL-17A↓	Regulating the gut microbiota disorders: the Simpson index and the relative abundance of *Akkermansia spp*. and *Corynebacterium spp*. are increased, and the relative abundance of the *Lachnospiraceae NK4A136* group are decreased	Chen et al. (2020b)
BALB/c mice	water pill	2.5 g/kg/d	Regulating inflammation and immune factors: Tcm cells↑, the balance of CD4^+^ Tem and CD8^+^ Tem cells is recovered; IL-2↓, IL-7↓, IL-12↓, IL-15↓, IL-10↑	Regulating the PI3K/Akt signaling pathway: the levels of PI3K, Akt, p-Akt, Id2, T-bet, FOXO3, Noxa, and C-myc proteins are decreased, and the levels of Rictor, Raptor, TSC1, TSC2, p-AMPKα, AMPKα, 4E-BP2, Kif2a and p70S6K are increased	Ge et al. (2020)
SD rat	water decoction	6g, 12 g and 24 g/kg/d	Regulating inflammatory factors: IL-1β↓, TNF-α↓	Inhibiting the TLR-2/NF-κB signaling pathway: the expression levels of TLR2, MyD88, IRAK4, and NF-κB p65 in the colon tissue are decreased	Zhaohua et al. (2022)
BALB/c mice	water pill	2.5 g/kg/d	Regulating immune cells: CD4^+^ Tcm↑, CD4^+^ mTfh cells↑, and the percentages of CD4^+^ and CD8^+^ expressions on central memory T cells are enhanced	Regulating the JAK/STAT5 signaling pathway: the levels of JAK1, PIAS3, STAT5, p-STAT5, BIM, BAX, caspase-3, and β-casein are decreased, and the levels of JAK3, PISA1, Bcl-2, and caveolin-1 are decreased	Wang et al. (2022a)
SD rat	water pill	5 g/kg/d	Regulating inflammation and oxidative stress factors: IFN-γ↓, IL-1β↓, IL-17↓, IL-4↓, calprotectin↓, MPO↓, MDA↓, NO↓, iNOS↓, T-AOC↑, SOD↑, eNOS↑	Inhibiting the ubiquitination of NEMO/NLK signaling pathway: the expressions of NF-κBp65, NLK, ubiquitinated NEMO and downstream proteins TAK, CYLD, P38 are decreased	Wang et al. (2019d)
SD rat	water pill	5 g/kg/d	Regulating inflammatory factors and enzyme activity of ATPase: TNF-α↓, IL-2↓, IL-15↓, sICAM-1↓, SDH↑, LDH↓, Na+K + -ATPase↑, Ca2+Mg2+-ATPase↑	Regulating the expression of wnt/β-catenin pathway related proteins:β-catenin, ubiquitination of Ub-NARF and Ub-TCF, and expression of Wnt/β-catenin downstream proteins are downregulated	Zhao et al. (2019)
BALB/c mice	water pill	2.5 g/kg/d	Regulating inflammatory factors and the differentiation of inflammatory dendritic cells: TNF-α↓, IL-1β↓, IL-6↓, IL-12p70↓, IL-10↑, iNOS + DCs↓, TNF-α+DCs↓, E-cadherin + DCs↓, MHC-II + DCs↓, GM-CSFR + DCs↓	1. Inhibiting TLR-4/NF-κB signaling pathway: the activation of the TLR4, MyD88, TRAF6, TAB2, and NF-κBp65 proteins and activated IκB are inhibited	Ge et al. (2022)
				2. Regulating the gut microbiota disorders: the enrichment of *Aerococcus* is inhibited, and the relative abundance of *norank f Lachnospiraceae*, *Lachnospiraceae UCG-006*, *Parvibacter*, *Akkermansia*, and *Rhodococcus* is increased	
BALB/c mice	water pill	2.5 g/kg/d	Regulating the differentiation of Tfh: Tfh10↑, Tfr↑, Tfh17↓, BCL-6+T cells↓, PD-1+ T cells↓, Blimp-1+ T cells↑	Activating the BCL-6/Blimp-1 signaling pathway: the expression of Bcl-6, STAT3 and p-STAT3 are inhibited, and the level of Blimp-1 is increased	Liu et al. (2020)
SD rat	water pill	5 g/kg/d	Improved the intestinal barrier integrity: Claudin-5↑, JAM1↑, VE-cadherin↑, and Connexin↑	Regulating PI3K/Akt and Rho/ROCK signaling pathways: the proteins expression levels of p-RhoA, ROCK1, PI3K, Akt, Notch1 and p-Rac are decreased, and levels of p-AMPKαand PTEN are decreased	Zhang et al. (2021c)
SD rat	water decoction	3.4 g/kg/d	Regulating inflammatory factors: IL-1β↓, TNF-α↓	Activating autophagy and restoring the balance between autophagy and apoptosis: the levels of LC3Ⅱ/Ⅰ and Beclin-1 are upregulated, and the number of autophagosomes is increased	Yu et al. (2024)
C57/BL mice	Freeze-dried powder	5 g/kg	Regulating inflammatory and apoptotic factors: TNF-α↓, Bax↓, Bcl-2↑, Bcl-2/Bax↑	Inhibiting p38 MAPK signaling pathway: the mRNA expressions of p38 MAPK, p53, caspase-3, c-jun, c-fos are decreased	Zhao et al. (2013)

Abbreviations: Akt: protein kinase B, AMPK: AMP-activated kinase, BAX: BCL-2, associated X, Blimp-1: B lymphocyte-induced maturation protein-1, BIM: Bcl-2-like protein 11, CREB: cAMP-response element binding protein, 4E-BP2: eukaryotic translation initiation factor 4E-binding protein 2, eNOS:endothelial nitric oxide synthase, FOXO3: forkhead box O3a, FT3: free triiodothyronine, FT4: free thyroxin, IL: interleukin, IFN-γInterferon gamma, iNOS:inducible nitric oxide synthase, IRAK: human interleukin-1, receptor-associated kinase, JAK: janus kinase, JAM-1: Junctional Adhesion Molecule-1, Kif2a: kinesin family member 2a, LDH: layered double hydroxide, p38MAPK: phosphorylated form of P38 mitogen activated protein kinase, MDA: malondialdehyde, MKK6: Mitogen-activated Protein Kinase Kinase 6, mTOR: mammalian target of rapamycin, MyD88: Myeloid differentiation primary response gene 88, NEMO: NF-κB, essential modulator, PD-1: programmed death-1, PD-L1: programmed death-ligand 1, PI3K: phosphatidylinositol 3-kinase, PTEN: phosphatase and tensin homolog deleted on chromosome ten, Rac1: ras-related C3 botulinum toxin substrate 1, RORγt: retinoic acid-related orphan receptor gamma t, ROCK: rho kinase, PPAR:peroxisome proliferator-activated receptor, p70S6K: 70-kDa ribosomal protein S6 kinase, PIAS: Protein inhibitors of activated STATs, ROS: reactive oxygen species, STAT: SATA: signal transducer and activator of transcription, SOCS: suppressor of cytokine signaling, SOD: superoxide dismutase, sICAM-1: Soluble intercellular adhesion molecule-1, TAB: Transforming Growth Factor β-Activated Protein Kinase 1 binding Protein, TRAF6: TNF, receptor associated factor 6, TLR: toll like receptor, T-AOC: total antioxidant capacity, TGF-β1: transforming growth factor-beta 1, TSC: tuberous sclerosis complex, Tfh: follicular helper T cell, Tfr: follicular regulatory T cells, Ub-NARF: ubiquitinated Nemo-like-kinase-associated ring finger protein, Ub-TCF: ubiquitinated T-cell factor.

### 3.2 Sishen Pill in the treatment of colon cancer

The RCT carried out by *Sun* et al. confirmed that the additional application of Sishen Pill in chemotherapy could significantly improve the patient’s discomfort symptoms, enhance the treatment effectiveness, reduce the probability of chemotherapy side effects (e.g., leukopenia, thrombocytopenia, liver and kidney function injury), and regulate immune cell levels (CD8^+^ cells↓, CD3^+^ and CD4^+^ cells↑) (Sun et al., 2021). Another clinical study revealed the therapeutic mechanism of the Sishen Pill in treating colon cancer from the perspective of gut microbiota. Researchers found that the richness and diversity of fecal microbiota in postoperative colon cancer patients were lower compared to healthy individuals; the Sishen Pill could improve this trend (Tan et al., 2023). For patients undergoing radical resection of colorectal cancer, Sishen Pill could not only alleviate clinical symptoms such as abdominal distension, tiredness, knee pain, waist acid, and cold but also reduce the tumor marker CEA and immune function indexes CD8^+^ and CD8^+^/CD4^+^ and increase the level of CD4^+^ (Zhang Y. et al., 2021). It should be pointed out that all three clinical studies mentioned above exist some methodological flaws, for example, not using a double-blind study design, and lacking relevant descriptions about allocation concealment, which to some extent reduces the reliability of clinical trial results; besides, these studies have not adopted the core indicators of clinical research on colon cancer, such as whether it can improve the survival rate of patients? Longer follow-up periods are necessary in the further research. The cell experiment on the molecular mechanism of Sishen Pill treating colon cancer showed that serum medicated with 10% Sishen Pill could downregulate the viability of HCT116 cells and the expression level of glucose transporter 1 (GLUT-1) and promote the activation of enzymes related to aerobic glycolysis, such as hexokinase and fructose-6-phosphate kinase. It could also decrease the overexpression of methyltransferase-like 3 protein and inhibit m^6^A RNA methylation, suggesting that Sishen Pill could regulate the glycolysis process by intervening in epigenetic modification, thereby inhibiting the proliferation of colon cancer cells (Jiang et al., 2023). In addition to the direct anticancer effect, *Cao* et al. also carried out research on the molecular mechanism of the Sishen Pill in inhibiting the transformation of colonic inflammatory lesions to colon cancer and found that the formula could downregulate the expression levels of nuclear factor e2-related factor 2 (Nrf2) and cyclooxygenase-2 (COX-2) in colon tissue, reducing the cancer formation rate of dextran sodium sulfate (DSS)-induced colitis mice; both oral and enema administrations had significant curative effects (Cao et al., 2012; Cao, 2013). In short, some studies also indicated the clinical effectiveness and molecular mechanism of Sishen Pill in the treatment of colon cancer; however, higher-quality clinical research and deeper mechanistic explorations are needed.

## 4 Research progress on metabolites of Sishen Pill in the treatment of IBD and colon cancer

### 4.1 Psoralea fructus

*Zhou* et al. studied the pharmacological effects and molecular mechanisms of psoralen, isopsoralen, and bakuchiol in treating IBD and confirmed that psoralen is the core pharmacodynamic substance and that the mechanism might be associated with the homeostasis of bile acids regulated by farnesoid X receptor (FXR)-fibroblast growth factor 15 (FGF15) pathways (Zhou, 2020). *Ami* et al. applied network pharmacology to identify 13 metabolites with good bioavailability after the oral administration of Psoraleae Fructus; and 11 metabolites could significantly reduce the overproduction of nitric oxide (NO), tumor necrosis factor- α (TNF-α) and interleukin-6 (IL-6) in macrophages induced by lipopolysaccharide (LPS) (Lee et al., 2023). Besides, network pharmacology analysis has also been used to explore molecular mechanism of isobavachalcone, an active metabolite of Psoralea Fructus, in the treatment of IBD, and *Yang* et al. confirmed AKT1, matrix metalloprotein 9 (MMP9), epidermal growth factor receptor (EGFR), insulin-like growth factor 1(IGF1), and steroid receptor coactivator (SRC) were its core targets (Yang et al., 2023a). The identification of effective metabolites is a key focus of botanical drug research, and the above studies is mainly based on the drug concentration in the blood, or bioavailability as the main screening criterion, which may inevitably overlook the indispensable effects of some metabolites with poor bioavailability, and they may exer intestinal protective effect by regulating the gut microbiota or microbial metabolites, rather than entering the peripheral loop. In addition, bakuchiol (Lim et al., 2019) and bavachin (Hung et al., 2019) have been shown to have ideal anti-inflammatory activities, and can they effectively treat IBD? Further research is needed for confirmation.

In recent years, the anticancer activity of various metabolites contained in Psoraleae Fructus has received widespread attention. For example, psoralen can inhibit the invasion and metastasis of human colon cancer HCT-116 cells, and its mechanism may be related to the downregulation of β-catenin, TCF4 proteins, their downstream target genes, vascular endothelial growth factor (VEGF), and MMP-9. Similarly, psoralidin was confirmed to reduce cell viability and enhance cell apoptosis by inhibiting the NF-κB and Bcl-2/BCL-2 associated X (Bax) signaling pathways (Jin et al., 2016); concurrently, it could also trigger oxidative damage-mediated apoptosis via rapidly boosting reactive oxygen species (ROS) generation (Sun et al., 2022). Bakuchiol can activate c-Jun N-terminal kinase (JNK) phosphorylation, induce ROS generation, and regulate the expression of death receptors and various anti-apoptotic proteins (Park et al., 2016). In addition, bavacin and 8-methoxypsoralen activate caspases by suppressing the MAPK and PI3K/AKT pathways, thereby promoting cancer cell apoptosis (Bartnik et al., 2017; Wang et al., 2023a) (see Table 2 for further details).

**TABLE 2:** Pharmacological effects and molecular mechanisms of the metabolites of Psoralea fructus in the treatment of IBD and colon cancer.

Disease	Category	Metabolites	Experimental model	Dosage	Pharmacological action	Molecular mechanism	References
IBD	coumarin	psoralen	C57BL/6	5,10,20 mg/kg	Regulating inflammatory factors: IL-6↓, IL-1β↓, TNF-α↓	Promoting he intestinal bile acid metabolism: the expression of Fxr, Fgf15 and some bile acid transporters are increased	Zhou (2020)
IBD	flavone	corylin	C57BL/6J	10,30,90 mg/kg	Regulating inflammatory factors: IL-6↓, TNF-α↓	Regulating the gut microbiota, tryptophan metabolism and 5-HT expression: 5-HT is reduced and 5-HTP is accumulated in the colon due to the binding of corylin and 5-HTDPC; besides, the concentration of tryptophan and the relative abundance of *Bacteroides*, *Escherichia-Shigella*, and *Turicibacter* is decreased and *Dubosiella*, *Enterorhabdus* and *Candidatus Stoquefichus* is increased	Wang et al. (2023f)
					Improving the intestinal barrier and blood-brain barrier: ZO-1↓, Occludin↓, Iba1↓(hippocampus)		
IBD	flavone	neobavaisoflavone	*In vivo*: C57BL/6J	*In vivo*: 30 mg/kg	Regulating immune cells: T_H_9 cell differentiation↓	Decreasing IL-9 production of CD4^+^ T cells by targeting PU.1: the expression of PU.1 (T_H_9-related transcription factors) is decreased	Guo et al. (2021a)
			*In vitro*: T helper 9 (T_H_9) cell	*In vitro*: 1 μmol/L			
colon cancer	coumarin	psoralen	HCT 116	20, 40 and 80 μg/mL	Inhibiting the cellular growth and metastasis: proliferation rate↓, migration rate↓, invasive ability↓	Inhibiting β-catenin/TCF4-MMP-9 signaling pathway: the levels of β-catenin, TCF4, VEGF and MMP-9 are decreased	Feng et al. (2021)
colon cancer	coumarin	psoralidin	SW480	5,10,20 μg/mL	Promoting the cellular apoptosis: cell viability↓, apoptosis rate↑, caspase-3 activity↑	Inhibiting the NF-κB and Bcl-2/Bax signaling pathways: the levels of NF-κB p65 and Bcl-2 protein expression are reduced, and Bax protein expression is increased	Jin et al. (2016)
colon cancer	coumarin	psoralidin	HT-29 and HCT-116	5,10,20 μg/mL	Promoting the cellular apoptosis: apoptosis cell rate↑, caspase 3/7 activity↑	Regulating the oxidative stress: the ROS generation is rapidly boosted and in turn triggering the DNA damage, mitochondria membrane potential decrease, and JUN 1/2 activation	Sun et al. (2022)
colon cancer	flavone	bavachin	*In vitro*: HT-29 and HCT 116	*In vitro*: 20, 30, 40 μmol/L^−1^	Promoting the cellular apoptosis: cell viability↓, apoptosis rate↑, cleaved PARP↑, cleaved Caspase-3↑	Up-regulating Gadd45a by activating the MAPK signaling pathway: the levels of Gadd45a and the phosphorylation levels of p38/ERK/JNK are upregulated	Wang et al. (2023a)
			*In vivo*: mouse xenograft model of human colorectal cancer	*In vivo*: 100 mg/kg/d			
colon cancer	monoterpene	bakuchiol	HCT116 and HT-29	1,5,10 μg/mL	Promoting the cellular apoptosis: DR4↑, DR5↑, cFLIP↓, Bcl2↓, XIAP↓, cleaved caspase-3, -8, -9 and PARP↑	Activating the ROS/JUN signaling pathway: the JNK phosphorylation is activated and the ROS generation is induced	Park et al. (2016)
colon cancer	coumarin	8-methoxypsoralen	SW 620	50, 100, 200 μg/mL	Promoting the cellular apoptosis: Bcl2↓, Bax↑, cleaved-3, -8, -9↑	Inhibiting the PI3K/AKT signalling pathway: the phosphorylation of AKT308 is decreased	Bartnik et al. (2017)

Abbreviations: Akt: protein kinase B, BAX: BCL-2, associated X, BCL: B-cell lymphoma, cFLIP: cellular fasassociated death domain-like interleukin-1β-converting enzyme-like inhibitory protein, DR: death receptor, ERK: extracellular regulated protein kinases, FXR: farnesoid X receptor, FGF15: fibroblast Growth Factor 15, 5-HTP: 5-hydroxytryptamine, Iba1:ionized calcium binding adaptor molecule 1, JUN: c-Jun N-terminal kinase, MAPK: mitogen activated protein kinase, MMP-9: Matrix metalloproteinase 9, NF-κB: nuclear factor-kappa B, PARP: Poly-ADP, ribose polymerase, PI3K: phosphatidylinositol 3-kinase, ROS: reactive oxygen species, TCF4: T cell factor 4, XIAP: X-linked inhibitor of apoptosis.

### 4.2 Myristica semen

Studies found that myristicin and linalool, two main metabolites in Myristica Semen, could exert anti-inflammatory and antioxidant effects by regulating the expression levels of NF-κ B and Nrf-2 (Tekeli et al., 2018; Ismail Abo El-Fadl and Mohamed, 2022). Compared to using diclofenac alone, the composite formulation of diclofenac and eugenol could better inhibit the nuclear translocation of NF-κB by activating the Nrf2/heme oxygenase-1 (HO-1) signaling pathway, thereby demonstrating better therapeutic effects against UC (Wang et al., 2023c). *Zhang* et al. developed a new phospholipid nanovesicle containing the volatile oil medicine eugenol to treat UC and confirmed that it was more conducive to percutaneous absorption and had better clinical efficacy (Zhang et al., 2020c).

In investigating Myristica Semen in treating colon cancer, *Chen* et al. used a network pharmacology method to screen nine active metabolites including galbacin and 24 core targets (Chen et al., 2023). Piras et al. (2012) identified the anticancer activity of essential oils and myristicin extracted from Myristicae Semen, confirming that they had a significant inhibitory effect on the growth of a colon cancer cell line (undifferentiated Caco-2 cells). In addition, Duan et al. (2020) showed that myristicin could inhibit the proliferation, migration, and invasion of colon cancer cells and induce cellular apoptosis by regulating the mitogen-activated protein kinase (MAPKK/MEK)/extracellular regulated protein kinase (ERK) signaling pathway. In addition, the regulatory effect of linalool on the oxidative response was beneficial for the treatment of colon cancer; it was confirmed that cellular apoptosis was induced by promoting the production of hydroxyl radicals and 4-HNE (a marker of oxidative stress due to increased lipid peroxidation) (Iwasaki et al., 2016). Moreover, some studies focused on the inhibitory effects of eugenol, isoeugenol, and dihydrodiiisoeugenol on colon cancer cells, showing that their anti-cancer mechanisms involve the regulation of metabolic pathways, apoptosis/metastasis-related gene expression, and the activation of endoplasmic reticulum stress-induced inhibition of autophagy (Li et al., 2021a; Ghodousi-Dehnavi et al., 2021; Bilgin et al., 2023). In above studies, Iwasaki et al. (Iwasaki et al., 2016) and Li et al. (Li et al., 2021b) used the tumor xenograft model in their experiments, while evidence from other studies mainly came from cell experiments. Generally speaking, the anti-tumor effects of botanical drugs are not single target or single pathway, and there exist complex interactions between different molecular pathways. It is crucial to explore the overall effects in tumor xenograft animal models, which greatly increases the credibility of research results. Further details are presented in Table 3.

**TABLE 3:** Pharmacological effects and molecular mechanisms of the metabolites of Myristicae semen in the treatment of IBD and colon cancer.

Disease	Category	Metabolites	Experimental model	Dosage	Pharmacological action	Molecular mechanism	References
IBD	phenylpropanoid	myristicin	SD rat	150 mg/kg/d	Regulating inflammatory factors, oxidative stress and ERS: TNF-α↓, IL-1β↓, COX-2↓, IL-10↑, SOD↑, GPx↑, MDA↓, MPO↓, ERS markers GRP78 and CHOP↓	Regulating NF-κB and Nrf-2/HO-1 signalling pathway: the expression levels of NF-κB are decreased, and Nrf-2 and HO-1 are increased	Ismail Abo El-Fadl and Mohamed (2022)
IBD	phenylpropanoid	linalool	Wistar rat	200 mg/kg/d	Regulating inflammatory factors and oxidative stress: MDA↓, IL-1β↓, IL-6↓,TNF-α↓, COX-2↓, CAT↑	Regulating the NF-κB and Nrf-2: the expression levels of NF-κB and COX-2 are decreased and the expression level of Nrf-2 are increased	Tekeli et al. (2018)
colon cancer	phenylpropanoid	myristicin	HCT116 and LOVO	2 and 5 μg/mL	Promoting the cellular apoptosis: survival rate↓, migration ability↓, invasion rate↓, apoptosis rate↓	Regulating the MEK/ERK signaling pathway: the expression level of E-cad is increased, and p-MEK1/2、p-ERK1/2、CyclinD1、MMP-2、MMP-9 are decreased	Duan et al. (2020)
colon cancer	phenylpropanoids	linalool	*In vitro*: HCT 116	*In vitro*: 1, 10, 100,250, 500, 1,000 μmol L^−1^	Promoting the cellular apoptosis: cell viability rate↓, cell apoptosis↑, tumor size and weight↓	Inducing the cancer-specific oxidative stress: the production of spontaneous hydroxyl radical is promoted, and 4-HNE, a marker of oxidative stress due to increased lipid peroxidation, is accumulated in the tumor tissue	Iwasaki et al. (2016)
			*In vivo*:SCID mice xenografted with human cancer cells	*In vivo*: 100 and 200 μg/kg^-1^			
colon cancer	phenylpropanoids	eugenol	HT-29 cell	500 μg ml^−1^	Inhibiting the gene expression related to cancer progression: APC and p53 genes↑, KRAS oncogene gene↓, cell survival percentage↓	Regulating metabolic pathways: (1) aminoacyl-tRNA biosynthesis; (2)valine, leucine, and isoleucine biosynthesis; (3)biotin metabolism; (4) steroid biosynthesis; (5)pantothenate and CoA biosynthesis; (6) glycerolipid metabolism; (7)galactose metabolism; and (8) glutamine and D-glutamate metabolism	Ghodousi-Dehnavi et al. (2021)
colon cancer	phenylpropanoids	isoeugenol	HT-29 cell	6.25,12.5,25,50,100 and 200 μg ml^−1^	Promoting the cellular apoptosis: cell viability↓, migration ability↓, Bax↑, p53↑, caspase-3,7,8,9↑ and the ratio of Bax/Bcl-2↑	Downregulating the expression of cell metastasis related genes: the mRNA expressions of MMP2, MMP9, VEGF and HIF-1αdecreased	Bilgin et al. (2023)
colon cancer	lignan	dehydrodiisoeugenol	*In vitro*: HCT 116 and SW620 cells	*In vitro*: 20, 40, and 60 μmol L^−1^	Inhibiting the cell growth: cell viability↓, cell inhibition rate↑, and the cell cycle arrest at the G1/S phase is induced	Activating endoplasmic reticulum stress-induced inhibition of autophagy via PERK/eIF2α and IRE1α/XBP-1s/CHOP pathways: protein expression levels of PERK,p-elF2α, IRE1α, XBP-1s and CHOP are increased	Li et al. (2021a)
			*In vivo*:CDX and PDX tumor xenograft model	*In vivo*: 40mg/kg^-1^			

Abbreviations: CAT: catalase activity, CHOP: CCAAT/enhancer-binding protein homologous protein, COX-2: cyclooxygenase-2, ERS: endoplasmic reticulum stress, eIF2α:eukaryotic translation initiation factor 2, ERK: extracellular regulated protein kinases, GPX: glutathione peroxidase, GRP78: glucose-related protein 78, HIF-1α: hypoxia-inducible factor 1α, HO-1: heme oxygenase, IRE1α: inositol-requiring enzyme 1α, IL:interleukin, MDA: malondialdehyde, MPO: myeloperoxidase, MEK: Ras/Raf/MAP, kinase-ERK, kinase, MMP: matrix metalloproteinase, NF-κB: nuclear factor-kappa B, Nrf-2: nuclear erythroid factor, SOD: superoxide dismutase, TNF-α: tumor necrosis factor-α, VEGF: vascular endothelial growth factor, XBP-1s: X Box Binding Protein-1.

### 4.3 Euodiae fructus

Studies suggested that NF-κB was the key target of evodiamine in the treatment of IBD, and downregulating NF-κB pathway proteins and inhibiting NOD like receptor heat protein domain related protein 3 (NLRP3) expression could alleviate inflammation-induced cell damage and repair the intestinal mucosal barrier (Shen et al., 2019; Ding et al., 2020). Simultaneously, evodiamine could also promote the regulation of the gut microbiota, especially by increasing the relative abundance of the beneficial bacterium *Lactobacillus* and intestinal acetate content, inhibiting the proliferation of the pathogenic *Escherichia coli*, and reducing plasma LPS and various inflammatory factor levels (Shen et al., 2019; Wang et al., 2020c; Ding et al., 2020). Another study suggested that kelch-like ECH-associated protein 1 (KEAP1) is a key target of rutecarpine, inhibiting the interactions between KEAP1 and Nrf2 by binding to the KEAP1 kelch domain, thereby activating Nrf2, promoting its nuclear translocation, upregulating the Nrf2-mediated antioxidant response, and achieving the pharmacological effect of improving intestinal mucosal injury (Zhang et al., 2020b).

In parallel, Chien et al. found that evodiamine could activate the MAPK signaling pathway and induce cell apoptosis and G2/M arrest by upregulating the phosphorylation levels of ERK and JNK proteins (Chien et al., 2014). Other researchers (Huang et al., 2015), Zhu (Zhu et al., 2021), and Zhang (Zhang et al., 2022) suggested that the anti-inflammatory effects of evodiamine also involved PI3K, STAT3, and NF-κB signaling pathways. In addition, evodiamine can reverse the epithelial-mesenchymal transition of tumor-associated fibroblasts induced by promoting the phosphorylation of Smad2 and Smad3, thereby reducing the migration and invasion abilities of tumor cells (Yang et al., 2019). In addition, Woong et al.’s research confirmed that rutecarpine could also inhibit wingless-type MMTV integration site family (Wnt)/β-catenin-mediated signaling pathway, thereby downregulating the expression levels of epithelial mesenchymal transition biomarkers such as MMP-7, Snail, and N-cadherin (Byun et al., 2022). Li et al. developed an EGFR targeting evodiamine-encapsulated polyamino acid nanoparticles to resolve the issue of low solubility and bioavailability and compared it with the traditional evodiamine formulation. The new preparation significantly increased the cytotoxicity of colon cancer cells and inhibited cell adhesion, invasion, and migration (Li et al., 2019a). Others have designed new metabolites based on evodiamine that have shown promising antitumor activity (Wang et al., 2020a; Li et al., 2020b). It should be pointed out that the dosage of evodiamine is still controversial in different studies. The minimum dose is 1 mg/kg, while the maximum dose is 40 mg/kg. It can be seen that a more detailed dose-response and dose-toxicity relationships of evodiamine needs to be further determined, which is crucial for guiding the clinical application. Further details are presented in Table 4.

**TABLE 4:** Pharmacological effects and molecular mechanisms of the metabolites of Euodiae fructus in the treatment of IBD and colon cancer.

Disease	Category	Metabolites	Experimental model	Dosage	Pharmacological action	Molecular mechanism	References
IBD	alkaloid	evodiamine	C57BL/6 mice	20, 40 and 80 mg/kg	Regulating inflammatory factors and oxidative stress: IL-6↓, IL-1β↓, TNF-α↓, MPO↓	1. Regulating NF-κB signal and NLRP3 inflammasome: the levels of p-p65, p-IκB, NLRP3, ASC, Caspase-1 and IL-1βare decreased	Shen et al. (2019)
						2. Regulating the gut microbiota and intestinal barrier: the expression levels of ZO-1 and occludin are increased, the concentration of LPS is decreased, and the abundance of *Escherichia coli* and *Lactobacillus* is re-balanced	
IBD	alkaloid	evodiamine	*In vitro*: Human THP-1 cells	*In vitro*: 10 μmol/L	Regulating inflammatory factors: IL-1β↓, IL-18↓	Inducing autophagosome-mediated degradation of inflammasome via inhibiting NLRP3 and NF-κB pathways: the protein expression level of P62 is decreased, and LC3-II is increased; meanwhile, expression levels of key pathway proteins NLRP3, cleaved-caspase-1, ASC, NF-κBp65 and IκB are decreased	Ding et al. (2020)
			*In vivo*: C57BL/6 mice	*In vivo*: 20, 40 and 60 mg/kg			
IBD	alkaloid	evodiamine	SD rat	20 mg/kg	Regulating inflammatory factors: TNF-α↓, IL-6↓, IL-1β↓, IL-10↑	Regulating the gut microbiota, intestinal barrier and circulating metabolite levels: the abundance of *Lactobacillus acidophilus*, the concentration of protective acetate production and the expression level of colonic claudin-1 is increased, and the levels of branched chain amino acids and aromatic amino acids are regulated	Wang et al. (2020c)
IBD	alkaloid	rutaecarpine	*In vitro*: HCT 116 cell and primary intestinal epithelial cell	*In vitro*: HCT 116 cell: 2.5, 5 and 10 μmol/L; primary intestinal epithelial cell: 10 μmol/L and 20 μmol/L	Regulating inflammatory factors: Cox2↓, Lcn2↓, TNF-α↓, IL-6↓	Inhibiting KEAP1-NRF2 interaction and upregulating NRF2-mediated antioxidant response: KEAP1 kelch domain is bound and NRF2 nuclear translocation is increased; meanwhile, H_2_O_2_-induced cytotoxicity and intracellular ROS accumulation are suppressed	Zhang et al. (2020b)
			*In vivo*: C57BL/6 mice	*In vivo*: 80 mg/kg			
IBD	terpenoid	limonin	*In vitro*: RAW 264.7	*In vitro*: 12.5, 25 and 50 pg/mL	Regulating inflammatory factors: TNF-α↓, IL-1β↓, IL-6↓, COX-2↓, iNOS↓	Inhibiting PERK-ATF4-CHOP pathway of ER stress and NF-κB signaling: expression levels of BIP, p-PERK, p-eIF2α, ATF-4, CHOP are decreased, and the nuclear translocation of NF-κB is inhibited	Song et al. (2021)
			*In vivo*: C57BL/6 mice	*In vivo*: 25,50,100 mg/kg			
IBD	terpenoid	limonin	*In vitro*: NCM460	*In vitro*: 2.5, 5, 10, 20, 40, 80 and 160 μg/mL	Regulating inflammatory factors: TNF-α↓, IL-6↓, IL-10↑	Regulating STAT3/miR-214 signaling pathway: expression levels of pSTAT3 and miR-214 are reduced and the expression levels of PTEN and PDLIM2 are restored	Liu S. et al. (2019)
			*In vivo*: C57BL/6 mice	*In vivo*: 40, 80 and 160 mg/kg			
colon cancer	alkaloid	evodiamine	HCoEpiC and CCD-18Co cells	16,160 and 320 nmol/L	Inhibiting the epithelial mesenchymal transition of colon epithelial cells: the tumour-associated fibroblasts-induced tumour-associated fibroblasts-like phenotype is reversed and their migration is inhibited	Mediating the expression of phosphorylated Smad2/3: the expression of ZEB1/Snail is downregulated, and the expression of phosphorylated Smad2/3 is upregulated, meanwhile, the ratios of pSmad2/Smad2 and pSmad3/Smad3 are increased	Yang et al. (2019)
colon cancer	alkaloid	evodiamine	COLO205 and HT-29 cells	2.5,5 and 10 μmol/L	Promoting the cellular apoptosis and G2/M arrest: cleaved-3 and -PARP↑, cycB1↑, cdc25c↑	Promoting the activation of MAPK signaling pathway: the protein phosphorylation levels of ERK and JNK are increased	Chien et al. (2014)
colon cancer	alkaloid	evodiamine	C57BL/6 mice	40 mg/kg	Regulating inflammatory factors and inhibiting the tumor development: TNF-α↓, IL-10↑, IL-6↓, IL-1β↓, the number and size of tumors↓	Regulating the gut microbiota and their metabolites: SCFAs-producing bacteria is enriched, the levels of the pro-inflammatory bacteria is reduced, and some microbiota metabolites (especially the tryptophan related metabolites) are regulated	Wang et al. (2021)
						Improving the intestinal barrier via multiple pathways: expression levels of occludin, ZO-1 and E-cadherin are increased, and some gene expressions of Wnt signaling pathway, Hippo signaling pathway and IL-17 signaling pathway are regulated	
colon cancer	alkaloid	evodiamine	*In vitro*: LoVo cells	*In vitro*: 0.25,0.5,1,2,4 μg/mL	Inhibiting the cell proliferation and promoting the cellular apoptosis: PCNA↓, apoptosis rate↑, caspase-3↑	Decreasing HIF-1α expression though IGF-1/PI3K/Akt signaling: the phosphorylation of Akt1/2/3, HIF-1αand IGF-1 are downregulated	Huang et al. (2015)
			*In vivo*: athymic nude mice	*In vivo*: 5, 10 and 20 mg/kg			
colon cancer	alkaloid	evodiamine	*In vitro*: HCT116 cells	*In vitro*: 0.5, 1 and 2 μg/mL	Promoting the cellular apoptosis: Bcl-2↓, Bad↑, apoptosis rate↑	Regulating BMP9 and HIF-1α/p53 signaling pathway: the expression levels of BMP9 and HIF-1αare upregulated and the phosphorylation of p53 is increased	Li et al. (2020a)
			*In vivo*: athymic nude mice	*In vivo*: 10 mg/kg			
colon cancer	alkaloid	evodiamine	HT29, HCT15 and SW480 cells	200 and 500 nmol/L	Inhibiting the cell proliferation and inducing G2/M arrest: number and volume of tumor ↓, G2/M accumulation↑	Suppressing the gene expression of controlling the proliferation of cancer stem cells: key genes of the Notch and Wnt signaling pathways are regulated	Kim et al. (2019)
colon cancer	alkaloid	evodiamine	SW 480 cells	5, 10 and 20 μg/mL	Promoting the cellular apoptosis: cell viability rate↓, cell apoptosis↑	Activating the autophagy: the protein expression levels of LC3 II and Beclin 1 are increased	Wang et al. (2019b)
colon cancer	alkaloid	evodiamine	*In vitro*: HCT116 cells	*In vitro*: 6 μmol/L	Inhibiting the tumor growth: survival ratio↓, number and volume of tumor↓	Regulating the gut microbiota: the relative abundance of *Campylobacter*, *Bifidobacterium* and *Lactobacillus* is increased, and *Enterococcus faecalis* and *Escherichia coli* are decreased	Zhu et al. (2021)
			*In vivo*: C57 mice	*In vivo*: 10 mg/kg		Downregulating the inflammatory IL6/STAT3/P65 signaling pathway: the expression of IL-6, p-STAT3, p-65 and the ratio of p-STAT3/STAT3 are decreased	
colon cancer	alkaloid	evodiamine	*In vitro*: HCT116 cells	*In vitro*: 0,1 and 5 μmol/L	Promoting the cellular apoptosis: cell viability↓, cleaved PARP↑, cleaved caspase-3↑	Targeting HSP70 and inactivating the HSP system: the N-terminal ATP-binding pocket of HSP70 is bound and causing its ubiquitin-mediated degradation	Hyun et al. (2021)
			*In vivo*: SCID mice xenografted with human cancer cells	*In vivo*: 20 mg/kg			
colon cancer	alkaloid	evodiamine	*In vitro*: SW480 cells	*In vitro*:100 and 200 μmol/L	Inhibiting inflammatory factors and inducing G2/M arrest: IL-1β↓, IL-2↓, IL-6↓, IL-17↓, IL-22↓, TNF-α↓, IL-15↑; G2/M accumulation↑	Inhibiting NF-κB signaling pathway: the phosphorylation levels of NF-κB, IKKα/β, IκBα and the expression level of S100a9 is decreased	Zhang et al. (2022)
			*In vivo*: C57BL/6	*In vivo*: 10 mg/kg			
colon cancer	alkaloid	evodiamine	*In vitro*: Lovo human colon cancer cells	*In vitro*: 7.5, 15, 30 and 60 μmol/L	Inducing the cellular apoptosis and S phase arrest: procaspase-3,8,9↓, caspase-3,8,9↑, Bax↑, Bcl-2/Bax ratio↓; cyclinA↓, cyclinB1↓, CDK1↓, CDK2↓,cdc25c↓	N/A	Zhang et al. (2010)
			*In vivo*: human colon carcinoma lovo xenograft mice	*In vivo*: 1 mg/kg			
colon cancer	alkaloid	evodiamine	*In vitro*: LoVo cells	*In vitro*:1,2,8 μg/mL	Inhibiting the cellular activity: cell viability, invasion and metastasis↓, tumor volume↓	Targeting EGFR protein to exert anti-tumor effects: protein expression levels of EGFR, VEGF, and MMP-2 are decreased	Li et al. (2019a)
			*In vivo*: BALB/c male athymic nude mice	*In vivo*: 4 mg/kg			
colon cancer	alkaloid	rutaecarpine	*In vitro*: RKO, SW480, HCT-15, HCT116, and Ls174T	*In vitro*: 5,10 and 20 μmol/L	Induced G0/G1 cell cycle arrest and apoptotic cell death: total cell death↓, migration rate↓, invasion rate↓, prolification rate↓, tumor volume and weight↓, G0/G1 accumulation↑	Inhibiting the Wnt/β-catenin-mediated signaling pathway: the expression levels of β-catenin and Wnt/β-catenin signaling pathway related proteins c-Myc, survivin, and cyclin D1 are downregulated; epithelial mesenchymal transition biomarkers such as MMP-7, Snail, and N-cadherin are downregulated	Byun et al. (2022)
			*In vivo*: xenograft nude mouse model	*In vivo*: 10 and 30 mg/kg			
IBD	terpenoid	limonin	Balb/c mice	50 mg/kg	Inhibit the initiation of colitis-associated-cancer: TNF-α↓, prostaglandin E2↓, tumor incidence/number↓	Regulated Nrf2, SOD2 and the immunophenotyping of lymphocytes: the expression levels of Nrf2 and SOD 2 are increased; T cells (CD4 and CD8) and B cells (CD19) in spleen tissues are increased, and the CD335 (natural killer cells) is restored to normal level	Ishak et al. (2021)

Abbreviations: ATF2: activating transcription factor 2, Akt: protein kinase B, ASC: apoptosis-associated speck-like protein containing CARD, BMP9: bone morphogenetic protein-9, CHOP: CCAAT/enhancer-binding protein homologous protein, COX-2: cyclooxygenase-2, EGFR: epidermal growth factor receptor, ERK: extracellular regulated protein kinases, eIF2α:eukaryotic translation initiation factor 2, HIF-1α: hypoxia-inducible factor 1α, HSP: heat shock protein, IGF-1: insulin-like growth factor 1, iNOS:inducible nitric oxide synthase, IL: interleukin, IκB: inhibitor of NF-κB, JNK: c-Jun N-terminal kinase, Keap1: Kelch-like ECH-associated protein 1, LPS: lipopolysaccharide, LCN2: lipocalin-2, MAPK: mitogen activated protein kinase, MPO: myeloperoxidase, NF-κB: nuclear factor-kappa B, NLRP3: NOD, like receptor heat protein domain related protein 3, N/A: not applicable, Nrf2: NF-E2-related factor 2, PI3K: phosphatidylinositol 3-kinase, PCNA: proliferating cell nuclear antigen, PARP: poly ADP-ribose polymerase, PERK: Protein kinase R (PKR)-like endoplasmic reticulum kinase, PTEN: phosphatase and tensin homolog, SATA: signal transducer and activator of transcription, SCFA: short-chain fatty acid, SCID: severe combined immunodeficient, SOD: superoxide dismutase, STAT: signal transducer and activator of transcription, S100a9: S11 calcium binding protein A9, TNF-α: tumor necrosis factor-α, VEGF: vascular endothelial growth factor, ZO-1: zonulin-1.

### 4.4 Schisandra chinensis

Multiple schisandrins have definite therapeutic effects in IBD. For example, schisandrin A (Wang et al., 2023b), schisandrin B (Liu et al., 2015), schisandrin C (Kim et al., 2022) and deoxyschizandrin (Yu and Qian, 2021) inhibited the NF-κB nuclear translocation and downstream pro-inflammatory signaling pathway activation; schisandrin B could also regulate AMPK/Nrf2, affect NLRP3 inflammasome, and then alleviate cell pyroptosis and intestinal epithelial damage caused by immune inflammation. In addition, Wang et al. conducted a pharmacokinetic analysis of seven different types of lignin in Schisandrae Chinensis and found that C_max_ and AUC_0-∞_ of schisandrin were significantly higher than those of other lignans; they also confirmed that it could treat UC by inhibiting the serum/glucocorticoid regulated kinase 1 (SGK1)/NLRP3 pathway and regulating the gut microbiota (Wang et al., 2023d).

Some studies focused on the therapeutic effects of metabolites in Schisandra Chinensis on colon cancer. Casarin et al. found that two types of lignins in Schisandrae Chinensis, (+)-deoxyschisandrin (1) and (−)-gomisin N, could induce the apoptosis of colon adenocarcinoma cells (LoVo); the mechanism was related to the downregulation of cyclin B protein expression, mediating G2/M phase arrest (Casarin et al., 2014). Schisandrin A has also been proven to have a regulatory effect on the cell cycle; it could downregulate the expression of HO-1 protein through the Nrf-2 signaling pathway, thereby reducing the production of reactive oxygen species and nitrogen oxides. However, it could also block NF-κB nuclear translocation and the activation of MAPKs to inhibit inflammatory response (Wan et al., 2019). Some studies focused on the therapeutic potential of Schisandrae Chinensis in inhibiting the “inflammation-cancer transformation.” Li et al.’s experiment confirmed that schisandrin B could inhibit the occurrence of colitis-associated cancer by regulating the gut microbiota and activating the phosphorylation of focal adhesion kinase and its downstream kinase (Li et al., 2019b). Pu et al. (2021) found that the pharmacological effect of schisandrin B in inhibiting the proliferation and metastasis of colitis-related tumors was related to the downregulation of silencing regulatory protein 1(SIRT1) and inducing the expression of smad ubiquitination regulatory factor 2 (SMURF2) (Pu et al., 2021). The anti-tumor activity of some non-specific active substances of Schisandrae Chinensis, such as citral, cannot be ignored either; the experiment by Sheikh et al. confirmed that citral could inhibit the proliferation of HCT116 and HT29 cells in a dose-dependent and time-dependent manner; its mechanism was related to mediating the phosphorylation of p53 protein and promoting the mitochondrial release of apoptogenic factors (Sheikh et al., 2017). See Table 5 for more details.

**TABLE 5:** Pharmacological effects and molecular mechanisms of the metabolites of Schisandra chinensis in the treatment of IBD and colon cancer.

Disease	Category	Metabolites	Experimental model	Dosage	Pharmacological action	Molecular mechanism	References
IBD	lignan	schisandrin A	SD rats	20, 40 and 80 mg/kg	Regulating the oxidative stress factors: GSH-P_X_↑, SOD↑, eNOS↑, iNOS↑, T-AOC↑; NO↓, MPO↓	N/A	Zhang et al. (2020a)
IBD	lignan	schisandrin A	Kunming mice	20, 40 and 80 mg/kg	The symptoms of colitis and intestinal inflammation are improved	Inhibiting NF-κB/COX-2 pathway: the mRNA and protein expression of NF-κB and COX-2 are decreased	Wang et al. (2023b)
IBD	lignan	schisandrin B	SD rat	20, 40 and 80 mg/kg	The symptoms of colitis and intestinal inflammation are improved	Regulating the expression level of RORγt and FoxP3: the protein and gene expression levels of FoxP3 increased, and RORγ are reduced	Chen and Chen (2018)
IBD	lignan	schisandrin B	*In vitro*: HCT-116 cells	*In vitro*: 40 μmol/L	Regulating inflammatory factors: TNF-α↓, IL-6↓, IL-1β↓, IFN-γ↓	Inhibiting NF-κB and MAPKs signal pathways: the expression levels of pIκBα, NF-κBp65, MAPK, JUN and ERK are decreased	Liu et al. (2015)
			*In vivo*: C57BL/6 mice	*In vivo*: 10 mg/kg			
IBD	lignan	schisandrin B	C57BL/6 mice	10, 40 and 100 mg/kg	Regulating inflammatory factors: TNF-α↓, IL-6↓, IL-18↓, IL-1β↓	Regulating the pyroptosis via AMPK/Nrf2/NLRP3 inflammasome: the protein expression level of NLRP3, pro-caspased and ROS-induced mitochondrial damage are decreased, and pAMPK/AMPK and Nrf2 are increased	Zhang et al. (2021a)
IBD	lignan	schisandrin B	*In vitro*: CACO2 and HCT116 cells	*In vitro*: 6.25 μmol/L and 12.5 μmol/L	1. Regulating inflammatory factors: TNF-α↓, IL-1β↓, IL-6↓,IL-12↓, IL-23↓	1. Activating FAK and its downstream signal: the ratio of p-FAK/FAK, p-JUN/JUN, p-P38/P38, p-AKT/AKT and p-ERK/ERK are increased	Li et al. (2019b)
			*In vivo*: C57BL/6 mice	*In vivo*: 15 and 30 mg/kg	2. Protecting the intestinal epithelial barrier: FITC-dextran permeabilization↓, E-cadherin↑, Occludin↑	2. Regulating the gut microbiota: the relative abundance of *Rhodospirillaceae*, *Mollicutes*, *Gastranaerophilales* and *Lachnospiraceae* is decreased, and the relative abundance of *Bacteroide*, *Rikenellaceae RC9* gut group, *Odoribacter laneus* YIT 12061 and *coprostanoligenes* is increased	
					3. Inhibit the initiation and promotion of colitis-associated-cancer		
IBD	lignan	schisandrin	C57BL/6 mice	20, 40 and 80 mg/kg	Regulating inflammatory factors: TNF-α↓, IL-1β↓, IL-18↓, IL-6↓	1. Inhibiting the SGK1/NLRP3 signaling pathway: the protein expression level of NLRP3, Caspase-1, SGK1 are decreased	Wang et al. (2023d)
						2. Regulating the gut microbiota: the relative abundance of *Lactobacilli* spp is increased, the relative abundance of *Bacteroides* decreased, and the conversion of primary bile acids to secondary bile acids is promoted	
IBD	lignan	schisandrin C	HT-29 and Caco-2 cells, intestinal organoid, *C. elegans* wild-type N2 strain	*In vitro*: 5, 10 and 20 μmol/L	Protecting the intestinal epithelial barrier: FITC-dextran permeabilization↓, MLCK and p-MLC↓, ZO-1↑, Occludin↑	Inhibiting NF-ĸB and p38MAPK/ATF2 pathways: the phosphorylation of NF-ĸB, p38 MAPK, and ATF2 are decreased, and the nuclear localization of NF-ĸB and ATF2 are inhibited	Kim et al. (2022)
				*In vivo*: 10, 25, 50 and 100 μmol/L			
IBD	lignan	deoxyschizandrin	SD rat	20, 40 and 80 mg/kg	Regulating inflammatory factors and oxidative stress factors: TNF-α↓, IL-1β↓, IL-6↓, SOD↑, MDA↓, CAT↑	Inhibiting the TLR4/NF-κB signaling pathway: the expression levels of TLR4, MyD88, and NF-κB are decreased	Yu and Qian (2021)
					Inhibiting the apoptosis: Caspase-3↓, Bax↓, Bcl-2↑		
colon cancer	lignan	schisandrin A	CRC cell lines DLD1, RKO, SW480, SW620 and normal human colon epithelial cell line CCD 841 CoN	50, 75, 100, and 150 μmol/L	Inducing the cellular apoptosis and G0/G1 phase arrest: p-Rb (S807/811)↓, Cyclin D1↓, Cdk4↓, Cdk6↓, cleaved-PARP↑, cleaved-Caspase3↑, Bcl-2↓	Inhibiting heat shock factor 1: the induction of HSF1 target proteins such as HSP70 and HSP27 inhibited	Chen et al. (2020a)
colon cancer	lignan	schisandrin A	HT 29 cells	0.25, 0.5 and 1 μmol/L	Regulating oxidative stress factors, inflammatory factors and inducing S and G2/M phase arrest: ROS↓, nitrite production↓, CAT↓, SOD↓, GPx↓, IL-8↓, S-phase and G2/M phase cell cycle arrest	Inhibiting Nrf-2/HO-1 signalling pathway, the translocation of NF-κB and the activation of MAPKs: the expression levels of HO-1, p-p38, p-ERK and p-JNK are decreased, the nuclear transcription of Nrf2 is activated and NF-κB is inhibited	Wan et al. (2019)
colon cancer	lignan	schisandrin B	SW480	20,40 and 80 μmol/L	Promoting the cellular apoptosis: proliferation inhibition rate↑, cell apoptosis↑, invasion rate↓	Regulating the p38MAPK signaling pathway: the protein expression levels of p-p38 and p-p53 are increased	Jiang et al. (2015)
colon cancer	lignan	schisandrin B	SW620	0.1, 1 and 10 mg/L	Inhibiting the cellular proliferation and migration:cellular activity↓, migration ability↓	Regulating the VEGF/PI3K/Akt signaling pathway: the expression levels of VEGFA、VEGF-R2、PI3K、Akt, p-Akt are decreased	Dai et al. (2018)
colon cancer	lignan	schisandrin B	*In vitro*: HCT 116 cells	*In vitro*: 3.125, 6.25, 12.5 and 25 μM	Promoting the cellular apoptosis: LDH activity↑, caspase-3/9 levels↑, E-cadherin↑, p53↑, Bax↑, MMP-9↓, β-catenin↓, COX-2↓, COX-1↓	Attenuating colitis-associated colorectal cancer through SIRT1 linked SMURF2 signaling: SMURF2 protein expression ia upregulated and SIRT1 is inhibited	Pu et al. (2021)
			*In vivo*: C57BL/6 mice	*In vivo*: 3.75, 7.5, 15 and 30 mg/kg			

Abbreviations: Akt: protein kinase B, ATF2: activating transcription factor 2, COX-2: cyclooxygenase-2, ERK: extracellular regulated protein kinases, eNOS:endothelial nitric oxide synthase, FAK: focal adhesion kinase, FoxP3: forkhead box protein P3, GSH-PX: glutathione peroxidase, HO-1: heme oxygenase, HSP: heat shock protein, IFN: interferon, IκB: inhibitor of NF-κB, iNOS:inducible nitric oxide synthase, JUN: c-Jun N-terminal kinase, MAPK: mitogen activated protein kinase, MPO: myeloperoxidase, MyD88: Myeloid differentiation primary response gene 88, MLCK: myosin light chain kinase, NF-κB: nuclear factor-kappa B, NO: nitric oxide, Nrf2: NF-E2-related factor 2, NLRP3: NOD, like receptor heat protein domain related protein 3, Nrf-2: nuclear erythroid factor, PI3K: phosphatidylinositol 3-kinase, RORγ: retinoic acid-related orphan receptor gamma t, ROS: reactive oxygen species, T-AOC: total antioxidant capacity, TLR4: toll-like receptor 4, SOD: superoxide dismutase,SGK1: serum/glucocorticoid regulated kinase 1, SIRT1: silencing regulatory protein 1, Smurf2: smad ubiquitination regulatory factor 2, VEGF: vascular endothelial growth factor.

## 5 Discussion on the common molecular mechanism of Sishen Pill in treating IBD and colon cancer

### 5.1 Regulating inflammation related signaling pathways

**FIGURE 2:**
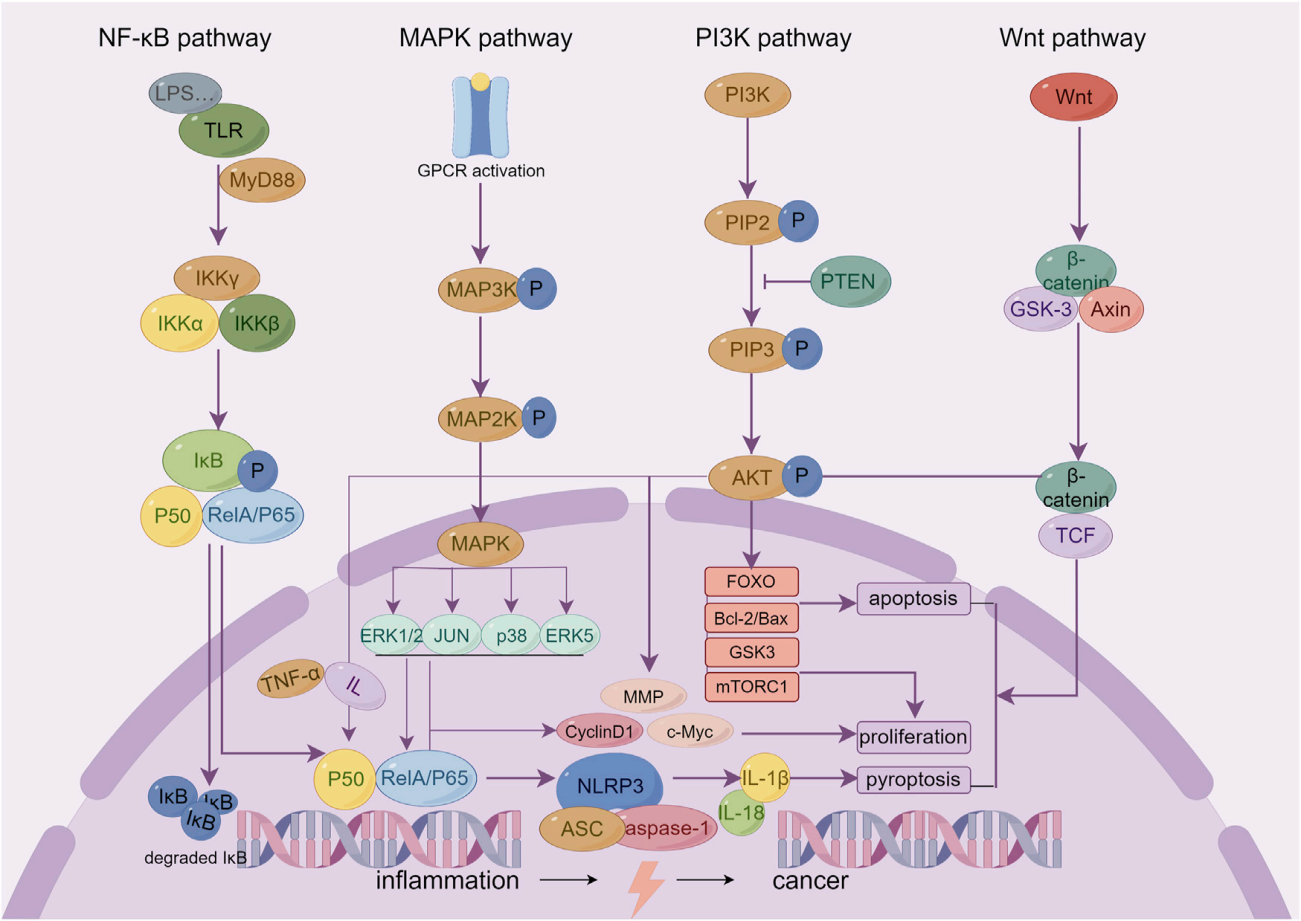
The key inflammatory related signaling pathways of Sishen Pill and its effective metabolites in treating IBD and colon cancer.

Chronic inflammation is not only an important feature of IBD but also a driver of the onset and development of colon cancer. Studies have found that chronic intestinal inflammation can cause DNA double chain breaks, oxidative stress damage, and epigenetic changes in intestinal epithelial cells, upregulate oncogenes, downregulate cancer suppressor genes, and promote the occurrence of dysplasia and cancer (Shah and Itzkowitz, 2022). Regulation of the inflammatory response is the core mechanism of Sishen Pill in treating IBD and inhibiting inflammation-cancer transformation, which involves multiple inflammation-related signaling pathways (Figure 2).

Many mechanistic studies on Sishen Pills focus on the regulatory effects of the NF-κB pathway (Wang et al., 2019d; Ge et al., 2022; Zhaohua et al., 2022). NF-κB is a classical key inflammatory modulator. LPS and other pro-inflammatory factors activate TLRs, induce NF-κB nuclear translocation, and regulate the gene expression of a variety of inflammatory mediators. In particular, LPS can promote the increase of TNF-α and multiple interleukins that act on macrophages to produce many inflammatory mediators and continuously induce NF-κB nuclear translocation to form a positive feedback cascade amplification effect. The basic activity of NF-κB is necessary for the normal proliferation and differentiation of cells to maintain the immune balance of epithelial tissue and inhibit the interference of inflammation on pithelial tissue homeostasis (Iacobazzi et al., 2023). Recently, a number of studies have focused on the role of NF-κB in promoting tumor cell apoptosis. As a landmark cell cycle protein, *cyclinD1* is also the target gene of NF-κB. The continuous activation of NF-κB can initiate cyclinD1 transcription, promote the G1/G0 phase-to-S phase transition, and lead to abnormal cell proliferation and cancer. Thus, inhibiting NF-κB activation or blocking its downstream key proteins is considered an important target for developing new antitumor drugs (Taniguchi and Karin, 2018; Deka and Li, 2023).

The pharmacological effects of the Sishen Pill in the treatment of IBD and colon cancer are also related to the regulation of the MAPK and PI3K/Akt signaling pathways. Extracellular regulated protein kinases (ERK)1/2, c-JNK, p38, and ERK5 are the main members of the MAPK family, and there is also extensive cross talk between different pathways: ERK mainly regulates cell growth and differentiation; JNK and p38 play more important roles in stress responses such as inflammation and cell apoptosis; and ERK5 can regulate pathological processes such as cell cycle acceleration and endothelial cell proliferation caused by growth factors and stress (Ronkina and Gaestel, 2022). In general, the MAPK signaling pathway can mediate the release of TNF-α, IL-1, IL-6, IL-8, and other inflammatory factors, cell apoptosis, and neutrophil activation, induce the expression of intracellular nitric oxide, improve the activity of intracellular inducible nitric oxide synthase, and induce the occurrence and development of IBD and colon cancer (Yong et al., 2009). In addition, PI3K is a key target that is closely related to inflammation and tumor development. In the inflammatory state, PI3K phosphorylates phosphatidylinositol 4,5-bisphosphate (PIP2) to generate phosphatidylinositol-3,4,5-triphosphate (PIP3), recruiting downstream proteins such as Akt. The activated AKT subsequently phosphorylates multiple downstream substrate proteins. PI3K can also promote the activation of NF-κB and regulate the inflammatory response by phosphorylating and inhibiting IκB kinase (IKK); it can also regulate biological processes such as cell proliferation, survival, apoptosis, and metabolism and then promote tumor progression. Briefly, it can 1) act on mammalian rapamycin target protein complex 1 (mTORC1) to promote protein synthesis and cell growth; 2) phosphorylate forkhead box O (FOXO) transcription factors, inhibit its transcriptional activity, and affect cell cycle, apoptosis, and metabolism; 3) inhibit the activity of glycogen synthase kinase 3 (GSK3) and regulate glycogen synthesis and cell cycle; 4) phosphorylate and activate the pro-apoptotic protein Bad, making it unable to bind to Bcl-2 or Bcl-XL, and thus reducing the occurrence of normal cell apoptosis (Mayer and Arteaga, 2016; Wang et al., 2023e). In the above study, Sishen Pill improved intestinal inflammatory factors, immune cell disorders, and a series of symptoms of IBD by downregulating the expression of key proteins in the MAPK (Zhao et al., 2013) and PI3K/Akt (Ge et al., 2020; Zhang et al., 2021c; Liu et al., 2021; Liu et al., 2022) signaling pathways, and its active metabolites bavachin (Wang et al., 2023a), myristicin (Duan et al., 2020), evodiamine (Chien et al., 2014), schisandrin B (Jiang et al., 2015), 8-methoxypsoralen (Bartnik et al., 2017), and schisandrin B (Dai et al., 2018). Consequently, the Sishen Pill could inhibit the progression of colon cancer by regulating the MAPK and PI3K pathways.

The NLRP3 inflammasome and Wnt signaling pathways affect pyroptosis and cell differentiation/apoptosis, respectively, and are potential targets for regulating colon inflammation-cancer transformation. NLRP3 inflammasome is composed of NLRP3, apoptosis-associated speck-like protein containing CARD (ASC) and effector pro-caspase-1, and can affect the occurrence and development of IBD and even cancer via regulating the maturation, secretion, and pyroptosis of IL-1β and IL-18. Studies found that for IBD patients during the active period, the production of IL-1β and IL-18 and the activity of caspase-1 increase, thereby mediating the occurrence of intestinal cell apoptosis (Qi et al., 2021). Cell apoptosis is an important pathological basis for the transformation from inflammation to cancer and can induce the release of pro-inflammatory cytokines and promote tumor infiltration into local tissues, thus increasing the risk of tumor occurrence and metastasis (He et al., 2024). In addition, the Wnt/β-catenin signaling pathway has been confirmed to influence the differentiation fate of cell development to a certain extent, affecting cancer cell proliferation, stemness, apoptosis, autophagy, and metabolism. The modification and degradation of β -catenin are key events in the occurrence and development of colon cancer (Zhao et al., 2022). The research indicates that Sishen Pills (Zhao et al., 2019) and their active metabolites, schisandrin B (Zhang et al., 2021a), schisandrin (Wang et al., 2023d), evodiamine (Kim et al., 2019; Shen et al., 2019; Ding et al., 2020), and rutaecarpine (Byun et al., 2022) could affect cell fate by regulating the NLRP3 and Wnt signaling pathways, thus offering a therapeutic role in IBD and colon cancer.

### 5.2 Inhibiting the oxidative stress

Research has shown that during chronic inflammation, innate immune cells such as macrophages produce large amounts of reactive oxygen species (ROS) and reactive nitrogen species (RNS), leading to the aggravation of oxidative stress. During the active phase of IBD, the expression of ROS in the intestinal mucosa increases, and the subsequent reaction of ROS with DNA can lead to chromosomal breakage, carcinogenesis, and tumor cell proliferation (Wu and Liu, 2022). Regulating the Nrf2/HO-1 pathway may be a way to treat IBD with Sishen Pills (Zhang et al., 2021b), involving their active metabolites such as myristicin (Ismail Abo El-Fadl and Mohamed, 2022), linalool (Tekeli et al., 2018), rutaecarpine (Zhang et al., 2020b) and schisandrin B (Zhang et al., 2021a). Under normal physiological conditions, the Nrf2 in cells binds to the kelch-like ECH-associated protein 1 (Keap1) in the cytoplasm and remains in a steady state; when Keap1 receives an oxidative stress signal, it can release Nrf2 and then transfer it to the nucleus and upregulate the expression of downstream antioxidant proteins, such as HO-1 (Ibrahim et al., 2023). At the same time, HO-1 can also block the NF-κB activation and downregulate the transcription of inflammatory factors and chemokines by inhibiting the production of cytokines and ROS (Wang and He, 2022); this may contribute to the treatment of IBD by inhibiting intestinal inflammation-cancer transformation (Lu et al., 2023).

**FIGURE 3:**
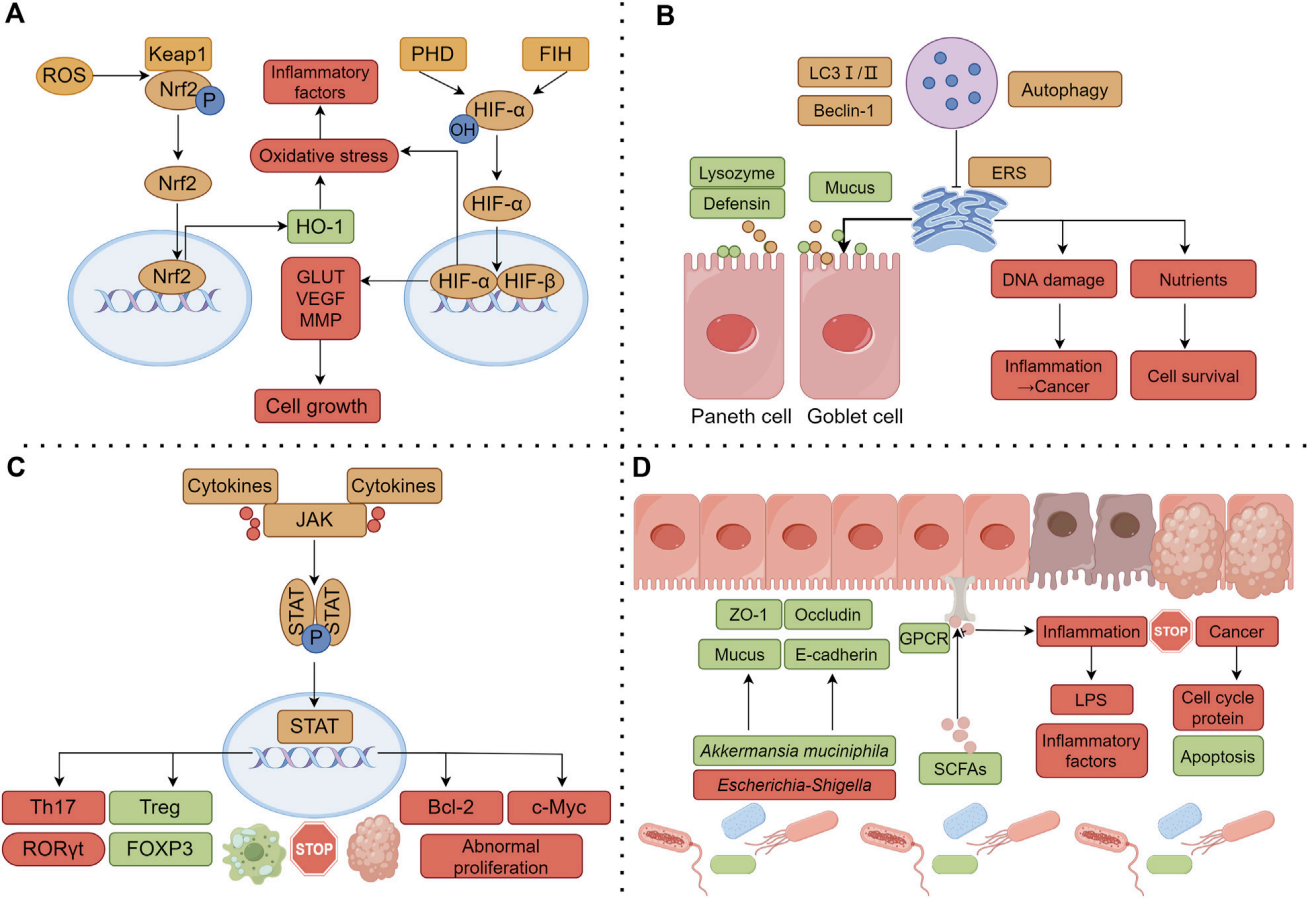
Common molecular mechanisms of Sishen Pill and its effective components in treating IBD and colon cancer. **(A)** Regulating the oxidative stress; **(B)** Regulating the mitochondrial autophagy; **(C)** Regulating intestinal immune cells; **(D)** Regulating the gut microbiota and internal barrier. Note: the green box represents the upregulated or promoted molecule, and the red box represents the downregulated or inhibited molecule.

As the α subunit of hypoxia inducible factor-1 (HIF-1), HIF-1α mediates the adaptive response of cells to a hypoxic environment. In general, HIF-1 is activated under cellular hypoxia; it can activate multiple target genes involved in regulating the cellular redox status to reduce ROS generation; it can also regulate the expression levels of mitochondria-specific genes to adapt to hypoxic environments and improve mitochondrial function. As the above studies showed, the anti-colon cancer effects of evodiamine (Huang et al., 2015; Li et al., 2020a) and isoeugenol may be mediated by HIF-1α. The activation of HIF-1α and its signaling pathway has bidirectional regulatory effects. Studies have confirmed that moderate activation can promote cell survival and increase the protective effect against injury stimuli, whereas excessive activation can aggravate damage to intestinal cells (Taylor and Colgan, 2007). Due to the abnormal vascular microenvironment and inadequate local blood and oxygen supply to the tumor, hypoxia is a common feature of colon cancer; HIF-1α in the activated state can supply energy to tumor cells by upregulating glucose transporters and glycolysis related enzymes, helping cells adapt to the hypoxic environment. Previous studies confirmed that regulating aerobic glycolysis is an important factor for Sishen Pill to treat colon cancer (Zhang et al., 2021d; Jiang et al., 2023); whether it is related to HIF-1α remains to be determined. In addition, the activation of HIF-1α can also promote the tumor angiogenesis and metastasis by up regulating the expression of VEGF and MMP. As the above studies showed, several effective metabolites of Sishen Pill, such as psoralen, isoeugenol, and evodiamine, can downregulate the expression of VEGF and MMP proteins (Li et al., 2019a; Feng et al., 2021; Bilgin et al., 2023), thus promoting the apoptosis and having a therapeutic effect in colon cancer (Figure 3A).

### 5.3 Regulating the mitochondrial autophagy

Research has shown that impaired autophagy can disrupt the function of intestinal epithelial cells and affect innate and adaptive immune responses, ROS production, and endoplasmic reticulum stress (ERS), ultimately promoting the occurrence or progression of IBD (Alula and Theiss, 2023). Antimicrobial peptides secreted by intestinal Paneth cells are an important component of the intestinal mucus layer; however, owing to autophagy dysfunction, patients would experience decreased secretion of defensins and lysozymes by Paneth cells, leading to a weakened ability of the intestinal mucosa to resist the colonization of bacteria in the gut, hindering bacterial clearance, and damaging the intestinal mucosal barrier (Cray et al., 2021). At the same time, autophagy also participates in the mucus secretion and degradation metabolism of goblet cells, maintaining a stable balance of interactions between the intestinal mucosa and the gut microbiota (Naama et al., 2023). In the pathological environment of IBD, sustained inflammatory stimulation can lead to protein imbalance and abnormal folding in the lumen of the endoplasmic reticulum, exacerbating ERS. Autophagy can reduce the negative effects of ERS by clearing abnormal proteins and damaged organelles. Previous studies found that the Sishen Pill (Yu et al.) and its metabolites, myristicin (Ismail Abo El-Fadl and Mohamed, 2022) and evodiamine (Ding et al., 2020) promote autophagy and exert a positive influence on downregulating intestinal inflammatory responses.

Other studies have shown that dehydrodiisoeugenol (Li et al., 2021a) and evodiamine (Wang et al., 2019b) promote tumor cell apoptosis and exert anti-colon cancer effects by activating autophagy. Current research suggests that autophagy has a dual role in cancer occurrence. Autophagy is a surveillance system in normal cells that removes damaged organelles and aggregated proteins through lysosomes, consequently reducing DNA damage and protecting cells from malignant transformation (Yamazaki et al., 2021). Clinical studies have shown that a lack of autophagy-related proteins such as LC-3II, ATG5, and Beclin 1 can indicate poor prognosis in colon cancer patients (Choi et al., 2014). Autophagy can also provide key nutrients for tumor growth and metabolism and support tumor formation by inhibiting apoptosis (Galluzzi et al., 2015). Autophagy plays different roles in the different stages of malignant tumor development; a deeper exploration of the pharmacological mechanism of inflammation-cancer transformation as a whole is needed (Figure 3B).

### 5.4 Regulating intestinal immune cells

The regulatory effects of Sishen Pill on intestinal immune cells are also important for inhibiting the progression of IBD inflammation and its transformation into colon cancer. Taking Tregs as an example, the number of Tregs in the inflammatory mucosa of patients with IBD often shows a compensatory increasing trend, but the degree of increase is insufficient to control mucosal inflammation, leading to a relatively insufficient state (Hovhannisyan et al., 2011). Peripheral blood cell analysis has shown that the number of Tregs decreases, and the number of pro-inflammatory Th17 cells increases in IBD patients (Eastaff-Leung et al., 2010). Animal experiments have shown that the adoptive transfer of Tregs can alleviate enteritis by inhibiting Th1 and Th17 inflammatory responses, further confirming the regulatory effects of Tregs on intestinal inflammation (Boschetti et al., 2017). Multiple studies have shown that Sishen Pill can upregulate Tregs while downregulating the proportion of Th17 cells, thereby inhibiting the progression of IBD (Liu et al., 2016; Wang et al., 2022b; Huang et al., 2022). Schisandrin B upregulates the expression of forkhead box protein P3 (FoxP3) and promotes Treg proliferation and differentiation, regulating the intestinal immunity of IBD (Liu et al., 2015). The possible role of Tregs in treating intestinal tumors has also received widespread attention, but there is still debate over how Tregs affect the occurrence and progression of tumors. Some studies suggest that Tregs can lead to tumor growth and deterioration by inhibiting anti-tumor immune responses (Saito et al., 2016), associated with poor prognosis of the disease; however, further research is needed to investigate the effect of the Sishen Pill on Tregs in colon cancer models.

Memory T cells (Tms) are a crucial part of inflammatory immune responses. Tms can usually be divided into three main groups: central memory T cells (Tcm), effective memory T cells (Tem), and tissue-resident memory T cells (Trm). Zhao et al. found that the specific activation of Tm can prevent the recurrence of Crohn’s disease (Zhao et al., 2020). Two studies found that Sishen Pills can increase the level of Tcm cells and inhibit intestinal inflammatory factors through the PI3K/Akt and JAK/STAT5 pathways (Ge et al., 2020; Wang et al., 2022a). In addition, Tcm has self-renewal and replication capabilities, can recognize tumor antigens, and exerts long-lasting anti-tumor effects (Wang et al., 2020d). However, there is currently no relevant report on the Sishen Pill and its active metabolites in treating colon cancer by regulating Tcm; a deeper understanding of the pharmacological mechanisms is urgently needed.

JAK/STAT is one of the central communication nodes of cell function and is essential for initiating innate immunity, coordinating adaptive immune mechanisms, and regulating inflammatory responses. In intestinal-associated lymphoid tissues, dendritic cells and other antigen-presenting cells initiate antigen-specific immune responses, determining the activation of B cells and the differentiation of initial T helper cells, driven by the cytokine-receptor interaction of JAK-STAT signaling. Furthermore, different subtypes of helper T cells (Th1, Th2, Th9, and Th17) regulate Tregs, macrophages, and dendritic cells, among other immune cells, thereby regulating the intestinal inflammatory response and inhibiting tumor occurrence (Hu et al., 2021). JAK/STAT pathway inhibitors have been used to treat IBD, showing good therapeutic potential in preclinical studies (Salas et al., 2020). As mentioned above, multiple studies have confirmed that Sishen Pill can regulate intestinal cellular immunity by regulating JAK-STAT and the expression of its downstream protein suppressor of cytokine signaling (SOCS), one of the key signaling pathways by which the pill inhibits IBD immune inflammation and inflammation-cancer transformation (Liu et al., 2016; Liu et al., 2020; Wang et al., 2022a; Kang et al., 2022) (Figure 3C).

### 5.5 Regulating the gut microbiota and intestinal barrier

Research has shown widespread dysbiosis in the gut microbiota of both IBD and colon cancer patients and that Sishen Pill can affect the integrity of the intestinal barrier by regulating the gut microbiota and its metabolites. The decrease of *Akkermansia muciniphila* (AKK) and the increase of *Escherichia-Shigella* are significant characteristics of the gut microbiota in IBD population (Morgan et al., 2012; Alam et al., 2016); animal experiments have shown that *Akk* can promote the production of intestinal mucus, regulate the expression of tight junction proteins, and reduce the expression levels of inflammatory and chemotactic factors in the colon and serum (Bian et al., 2019). In addition, oral administration of inactivated *Akk* or the outer membrane protein of *Akk* (Amuc_1100) can also regulate CD8^+^ T cells, improving IBD and preventing the occurrence of CACC (Wang et al., 2020b). Another study suggested that AKK bacteria could enrich M1-like tumor associated macrophages in the colon cancer microenvironment in NLRP3 dependent way, thereby inhibiting tumor formation and development (Fan et al., 2021a). On the contrary, certain types of *Escherichia-Shigella* can escape host immunity, adhere to and invade intestinal epithelium and macrophages in hosts with genetic susceptibility to IBD, and initiate IBD development (Zangara et al., 2023). Also, the genotoxin produced by *Escherichia-Shigella* can penetrate the colon cell membrane and migrate to the nucleus, causing DNA double strand breaks, cell cycle arrest, chromosomal aberrations, intestinal epithelial damage, and eventually leading to cancer (Fan et al., 2021b). The above studies show that Sishen Pill can increase the relative abundance of *AKK* in the intestine (Chen et al., 2020b; Ge et al., 2022; Jin et al., 2023) and its effective metabolites, evodiamine, and corylin, leading to significant inhibition of *Escherichia-Shigella* the proliferation (Zhu et al., 2021; Wang et al., 2023f) thereby exerting anti-inflammatory and anticancer pharmacological activities.

Some metabolites of the gut microbiota, such as short-chain fatty acids (SCFAs), are also important for developing colitis and tumors. Studies have shown a decreasing trend in intestinal SCFAs in both IBD and colon cancer populations (Wang et al., 2019c; Dalile et al., 2019). As the main source of energy for intestinal epithelial cells, SCFAs not only promote the proliferation and differentiation of intestinal epithelial cells, reduce cell apoptosis, and maintain the mechanical barrier of the intestinal mucosa but also improve the secretion of intestinal mucoproteins, lubricate the intestine, block the adhesion of pathogens to the intestinal mucosa, and inhibit the occurrence of intestinal immune inflammation (Sun et al., 2017). In addition, butyrate in SCFAs has been proven to promote apoptosis and inhibit the proliferation of human colon cancer cells by activating G-protein coupled receptor 109A (GPR109A) (Moniri and Farah, 2021). The above research indicates that while regulating the gut microbiota, Sishen Pill can increase the contents of total SCFAs and butyrate in the intestine, thereby improving the inflammatory microenvironment of the intestine (Wang et al., 2022b).

Gut microbiota can affect the morphology and function of the intestinal barrier through various pathways; the integrity of the intestinal barrier is of great significance for the treatment of IBD and colon cancer. Post et al. detected 28 mucin proteins in the colonic mucosa of UC patients and found that seven mucin proteins, such as mucin 2 (MUC2), were significantly reduced; 30% of UC patients had abnormal permeability of the mucus layer, suggesting that abnormal colonic barrier function promotes the occurrence of UC (van der Post et al., 2019). Rath et al. showed that healing of the intestinal barrier has a high predictive value for the course of patients with remission-phase IBD; predictive ability of intestinal barrier healing might far exceed established or emerging parameters, such as endoscopic and histological remission (Rath et al., 2023). In addition, intestinal barrier damage and microbial translocation can activate chronic inflammation, further promoting the secretion of pro-inflammatory factors by immune cells and accelerating the process of colonic inflammation-cancer transformation (Shalapour and Karin, 2020). Another study found that when the intestinal vascular barrier is damaged, intestinal bacteria are more likely to spread to the liver, promoting the formation of a pre-metastatic niche for “colon-liver” metastasis, thereby promoting the recruitment of metastatic cells (Bertocchi et al., 2021). As mentioned earlier, Sishen Pills (Zhang et al., 2021c), schisandrin B (Li et al., 2019b), schisandrin C (Kim et al., 2022), and corylin (Wang et al., 2023f) can regulate the secretion of intestinal epithelial tight junction proteins and mucin, repair damaged intestinal mucosal barriers and inhibit the progression of IBD (Figure 3D).

## 6 Limitations and outlook

As a classic proprietary Chinese medicine for treating diarrhea, the curative effect of the Sishen Pill on IBD and colon cancer has been widely studied. Briefly, TCM formulas can regulate multiple targets simultaneously and exert integrated pharmacological effects; they can not only regulate intestinal immune inflammation disorders and fight against tumors but can also improve various symptoms, such as abdominal pain and diarrhea, enhancing the patient’s quality of life. In addition, preventive treatment of disease is a characteristic and an advantage of TCM; the application of the Sishen Pill in the early stage of IBD can effectively inhibit the transformation from inflammation to colon cancer. In summary, based on Western medical treatment, accumulating evidence suggests that the use of TCM represented by Sishen Pills can often bring more clinical benefits to patients. However, in terms of the current research on Sishen Pills, many limitations still need to be addressed.

Firstly, the above mentioned clinical studies and experimental studies have preliminarily confirmed the evidence that Sishen Pill can effectively treat IBD and colon cancer, however, the standardization of study design and reporting still needs further improvement. For example, 1) most studies do not provide quality testing reports and specific preparation methods of Sishen Pill; 2) there is a lack of description in the report regarding the experimental design methods and bias control strategies, such as specific measures for randomization of groups and baseline data of different groups before intervention; 3) there is a lack of description of animal or cell model selection criteria and modeling methods. In future studies, we recommend that: 1) design and report rigorously according to the requirements of the Cochrane Handbook (clinical study) (PT and Sally, 2024) and ARRIVE guidelines (animal experiments) (Kilkenny et al., 2012); 2) clinical studies should adopt internationally recognized major outcome measures, and basic experiments are necessary to observe the overall therapeutic effect of Sishen Pill on experimental animals, rather than just cell experimental evidence.

Second, there is still insufficient evidence on the safety of the Sishen Pill, posing a hidden danger in its clinical application. In recent years, liver damage caused by Psoraleae Fructus has become a focus of attention (Xu and Xiao, 2023). *Guo* et al. confirmed that the mechanism of liver injury could be associated with oxidative stress and mitochondrial damage-mediated apoptosis (Guo et al., 2021b) and that it may also be involved in liver regeneration, bile metabolism, energy metabolism, and other processes (Fan et al., 2024; Feng et al., 2024). Compared to bakuchiol, psoralen and isopsoralen have been confirmed to have stronger liver toxicity *in vivo*; their toxic effects are positively correlated with dosage (Mu et al., 2018). Similarly, Euodiae Fructus has also been shown to pose a potential risk of liver injury (Kong et al., 2023); its mechanism of action may be related to peroxidation injury, inflammatory factor mediation, mitochondrial damage, and drug‒ protein adduct formation (Wei et al., 2020). Other studies have reported that evodiamine exerts potential nephrotoxicity and cardiotoxicity (Yang et al., 2021). The impact of Myristicae Semen on the liver is two-sided; some studies confirmed that myristicin has a protective effect on drug-induced liver injury (Sohn et al., 2008; Yimam et al., 2016; Yang et al., 2018), while others found that Myristicae Semen extracts can also damage liver cells, increase serum transaminase levels and that the toxic effects are time- and dose-dependent (Cao et al., 2020). We believe that the “dose-effect-toxicity” relationship of the Sishen Pill should be further clarified through basic research to evaluate the clinical efficacy and safety. Further research should be conducted to enhance efficacy and detoxification using methods such as the processing and rational compatibility.

Third, although current research suggests that multiple metabolites in Sishen Pill have therapeutic effects on IBD and colon cancer, the active metabolites of this formula still need to be clarified. The currently elucidated molecular mechanisms may provide important links to its integrated pharmacological effects; however, the most critical target of action and differential pathways among different metabolites still require further exploration. We suggest using a more precise research approach as the next step in basic research. Luo and others (Luo et al., 2022) confirmed that MyD88 is the specific target for the anti-inflammatory effects of schisandrin B using target knockout models, carrying out target-metabolites binding assays, molecular docking, and experimental verification. In future research, high-throughput screening of the proteome and target-metabolites binding assays, vital for determining deeper pharmacological mechanisms of action and revealing the scientific connotations of TCM compatibility, should be widely used.

Fourth, clinical evidence of the Sishen Pill in treating colon cancer and inhibiting inflammation-cancer transformation is still lacking. As mentioned above, there are relatively few clinical and basic studies of Sishen Pill on treating colon cancer; more evidence is needed on its effective metabolites. However, there are complex interactions between different chemical metabolites, so the efficacy evaluation and mechanism exploration of Sishen Pill in treating colon cancer need to be further carried out. Besides, the transformation of colonic “inflammation-cancer” is a dynamic process; at present, only a few studies have focused on the effect of Sishen Pill and its metabolites on CACC. Specific clinical application strategies, including the best application nodes and treatment courses, are also urgently needed. In addition to the traditional dosage forms, the efficacy and safety of decoction enemas and volatile oils have also been preliminary confirmed; however, the differences in the indications of different dosage forms and how better market transformations can be performed need to be addressed stepwise through a series of studies. Large sample sizes, long-term follow-up RCTs, and real-world post-marketing reevaluations of formulas are necessary to better address the above issues.

## 7 Conclusion

Sishen Pills and its metabolites show great potential in the treatment of IBD, colon cancer and the inhibition of colonic inflammation-cancer transformation. Modern pharmacological research has confirmed that Sishen Pills molecular mechanisms mainly involve regulating inflammatory signaling pathways, inhibiting oxidative stress, improving mitochondrial mitophagy, regulating intestinal immune cells, and modulating the gut microbiota. Meanwhile, we should not overlook the limitations of current research. Due to the lack of rigorously designed large-sample RCTs, it is still difficult to answer questions about the long-term effectiveness and safety of the Sishen Pill in treating IBD and colon cancer. In future research, we recommend combining RCTs with real-world clinical studies to obtain stronger clinical evidence. At the same time, it is important to strengthen research on potential core metabolites of Sishen Pill, such as evodiamine and schisandrins, and clarify their key molecular targets, in order to lay the foundation for the new drug development.

## References

[B1] Alam A. Leoni G. Quiros M. Wu H. Desai C. Nishio H. (2016). The microenvironment of injured murine gut elicits a local pro-restitutive microbiota. Nat. Microbiol. 1, 15021. 10.1038/nmicrobiol.2015.21 27571978 PMC5076466

[B2] Alula K. M. Theiss A. L. (2023). Autophagy in Crohn's disease: converging on dysfunctional innate immunity. Cells 12 (13), 1779. 10.3390/cells12131779 37443813 PMC10341259

[B3] Bartnik M. Sławińska-Brych A. Żurek A. Kandefer-Szerszeń M. Zdzisińska B. (2017). 8-methoxypsoralen reduces AKT phosphorylation, induces intrinsic and extrinsic apoptotic pathways, and suppresses cell growth of SK-N-AS neuroblastoma and SW620 metastatic colon cancer cells. J. Ethnopharmacol. 207, 19–29. 10.1016/j.jep.2017.06.010 28627461

[B4] Bertocchi A. Carloni S. Ravenda P. S. Bertalot G. Spadoni I. Lo Cascio A. (2021). Gut vascular barrier impairment leads to intestinal bacteria dissemination and colorectal cancer metastasis to liver. Cancer Cell 39 (5), 708–724.e11. 10.1016/j.ccell.2021.03.004 33798472

[B5] Bian X. Wu W. Yang L. Lv L. Wang Q. Li Y. (2019). Administration of Akkermansia muciniphila ameliorates dextran sulfate sodium-induced ulcerative colitis in mice. Front. Microbiol. 10, 2259. 10.3389/fmicb.2019.02259 31632373 PMC6779789

[B6] Bilgin S. Erden Tayhan S. Yıldırım A. Koç E. (2023). Investigation of the effects of isoeugenol-based phenolic compounds on migration and proliferation of HT29 colon cancer cells at cellular and molecular level. Bioorg Chem. 130, 106230. 10.1016/j.bioorg.2022.106230 36375352

[B7] Bonovas S. Fiorino G. Lytras T. Nikolopoulos G. Peyrin-Biroulet L. Danese S. (2017). Systematic review with meta-analysis: use of 5-aminosalicylates and risk of colorectal neoplasia in patients with inflammatory bowel disease. Aliment. Pharmacol. Ther. 45 (9), 1179–1192. 10.1111/apt.14023 28261835

[B8] Boschetti G. Kanjarawi R. Bardel E. Collardeau-Frachon S. Duclaux-Loras R. Moro-Sibilot L. (2017). Gut inflammation in mice triggers proliferation and function of mucosal Foxp3+ regulatory T cells but impairs their conversion from CD4+ T cells. J. Crohns Colitis 11 (1), 105–117. 10.1093/ecco-jcc/jjw125 27364948

[B9] Byun W. S. Bae E. S. Kim W. K. Lee S. K. (2022). Antitumor activity of rutaecarpine in human colorectal cancer cells by suppression of wnt/β-catenin signaling. J. Nat. Prod. 85 (5), 1407–1418. 10.1021/acs.jnatprod.2c00224 35544614

[B10] Cao Y. (2013). Preventive effect of traditional Chinese medicine "Si-Shen bolus" on colitis associated cancer in mice. Doctor. master’s thesis. Liaoning: Liaoning University of Traditional Chinese Medicine.

[B11] Cao Y. Wang Y.-J. Xie X. Tian Z.-G. (2013). Effect of Sishen Pill on the expression of CD133 protein in colon cancer induced by colitis in mice. Chin. Med. Mod. Distance Educ. China 11 (08), 145–146.

[B12] Cao Y. Zhao D.-Y. Chai J.-Y. Tian Z.-G. (2012). Chemopreventive effect of sishen pill on experimental colon cancer in rats. J. Liaoning Univ. Traditional Chin. Med. 14 (11), 127–129. 10.13194/j.jlunivtcm.2012.11.129.caoy.063

[B13] Cao Z. Xia W. Zhang X. Yuan H. Guan D. Gao L. (2020). Hepatotoxicity of nutmeg: a pilot study based on metabolomics. Biomed. Pharmacother. 131, 110780. 10.1016/j.biopha.2020.110780 33152938

[B14] Casarin E. Dall'Acqua S. Smejkal K. Slapetová T. Innocenti G. Carrara M. (2014). Molecular mechanisms of antiproliferative effects induced by Schisandra-derived dibenzocyclooctadiene lignans (+)-deoxyschisandrin and (-)-gomisin N in human tumour cell lines. Fitoterapia 98, 241–247. 10.1016/j.fitote.2014.08.001 25110194

[B15] Cassotta M. Cianciosi D. De Giuseppe R. Navarro-Hortal M. D. Armas Diaz Y. Forbes-Hernández T. Y. (2023). Possible role of nutrition in the prevention of inflammatory bowel disease-related colorectal cancer: a focus on human studies. Nutrition 110, 111980. 10.1016/j.nut.2023.111980 36965240

[B16] Chen B. C. Tu S. L. Zheng B. A. Dong Q. J. Wan Z. A. Dai Q. Q. (2020a). Schizandrin A exhibits potent anticancer activity in colorectal cancer cells by inhibiting heat shock factor 1. Biosci. Rep. 40 (3). 10.1042/bsr20200203 PMC7069920 32110802

[B17] Chen F. Yin Y. T. Zhao H. M. Wang H. Y. Zhong Y. B. Long J. (2020b). Sishen pill treatment of DSS-induced colitis via regulating interaction with inflammatory dendritic cells and gut microbiota. Front. Physiol. 11, 801. 10.3389/fphys.2020.00801 32754049 PMC7381313

[B18] Chen L.-L. Chen R.-J. (2018). Therapeutical effect of schizandrin B on ulcerative colitis in rats and underlying mechanism. China Pharm. 21 (11), 1941–1945.

[B19] Chen X. Xu H. Li R.-N. Li X.-L. Zhao L.-N. Xu Z.-L. (2023). Mechanism of Myristica fragrans Houtt.Against colorectal carcinoma based on network pharmacology and molecular docking. J. Med. Inf. 36 (14), 16–21.

[B20] Chen Y. Chen L. Xing C. Deng G. Zeng F. Xie T. (2020c). The risk of rheumatoid arthritis among patients with inflammatory bowel disease: a systematic review and meta-analysis. BMC Gastroenterol. 20 (1), 192. 10.1186/s12876-020-01339-3 32552882 PMC7301504

[B21] Chien C. C. Wu M. S. Shen S. C. Ko C. H. Chen C. H. Yang L. L. (2014). Activation of JNK contributes to evodiamine-induced apoptosis and G2/M arrest in human colorectal carcinoma cells: a structure-activity study of evodiamine. PLoS One 9 (6), e99729. 10.1371/journal.pone.0099729 24959718 PMC4069003

[B22] Choi J. H. Cho Y. S. Ko Y. H. Hong S. U. Park J. H. Lee M. A. (2014). Absence of autophagy-related proteins expression is associated with poor prognosis in patients with colorectal adenocarcinoma. Gastroenterol. Res. Pract. 2014, 179586. 10.1155/2014/179586 24723943 PMC3960741

[B23] Chopra B. Dhingra A. K. Dhar K. L. (2013). Psoralea corylifolia L. (Buguchi) - folklore to modern evidence: review. Fitoterapia 90, 44–56. 10.1016/j.fitote.2013.06.016 23831482

[B24] Cray P. Sheahan B. J. Dekaney C. M. (2021). Secretory sorcery: Paneth cell control of intestinal repair and homeostasis. Cell Mol. Gastroenterol. Hepatol. 12 (4), 1239–1250. 10.1016/j.jcmgh.2021.06.006 34153524 PMC8446800

[B25] Dai G.-L. Gong T. Li Y. Ding K. Wu K.-L. Li Z.-W. (2018). Effect of schisandrin B on proliferation and migration of human SW620 colon cancer cell via VEGF/PI3K/Akt signaling pathway. Chin. Pharm. J. 53(14)**,** 1186–1191.

[B26] Dalile B. Van Oudenhove L. Vervliet B. Verbeke K. (2019). The role of short-chain fatty acids in microbiota-gut-brain communication. Nat. Rev. Gastroenterol. Hepatol. 16 (8), 461–478. 10.1038/s41575-019-0157-3 31123355

[B27] D'Ascenzo F. Bruno F. Iannaccone M. Testa G. De Filippo O. Giannino G. (2023). Patients with inflammatory bowel disease are at increased risk of atherothrombotic disease: a systematic review with meta-analysis. Int. J. Cardiol. 378, 96–104. 10.1016/j.ijcard.2023.02.042 36863421

[B28] Deka K. Li Y. (2023). Transcriptional regulation during aberrant activation of NF-κB signalling in cancer. Cells 12 (5), 788. 10.3390/cells12050788 36899924 PMC10001244

[B29] Ding W. Ding Z. Wang Y. Zhu Y. Gao Q. Cao W. (2020). Evodiamine attenuates experimental colitis injury via activating autophagy and inhibiting NLRP3 inflammasome assembly. Front. Pharmacol. 11, 573870. 10.3389/fphar.2020.573870 33240089 PMC7681073

[B30] Duan C.-Y. He N.-N. Zhu L. Fan L. Yi F. Wang T. (2020). The mechanism of myristicin lnhibiting proliferation, migration and invasion of colon cancer cell lines. Mod. Traditional Chin. Med. Materia Medica-World Sci. Technol. 22 (04), 907–913.

[B31] Eastaff-Leung N. Mabarrack N. Barbour A. Cummins A. Barry S. (2010). Foxp3+ regulatory T cells, Th17 effector cells, and cytokine environment in inflammatory bowel disease. J. Clin. Immunol. 30 (1), 80–89. 10.1007/s10875-009-9345-1 19936899

[B32] Fan B.-B. Zhong R.-Z. Ma Z. Han Y. Shu T. (2024). Progress in pharmacological studies of buguzhi (Psoralea). Chin. Archives Traditional Chin. Med., 1–8.

[B33] Fan L. Xu C. Ge Q. Lin Y. Wong C. C. Qi Y. (2021a). A. Muciniphila suppresses colorectal tumorigenesis by inducing TLR2/NLRP3-mediated M1-like TAMs. Cancer Immunol. Res. 9 (10), 1111–1124. 10.1158/2326-6066.Cir-20-1019 34389559

[B34] Fan X. Jin Y. Chen G. Ma X. Zhang L. (2021b). Gut microbiota dysbiosis drives the development of colorectal cancer. Digestion 102 (4), 508–515. 10.1159/000508328 32932258

[B35] Feng K.-R. Wu Y.-L. Li W.-X. Wang X.-Y. Zhang H. Yang L.-G. (2024). Research progress on hepatotoxicity of Psoralea and its attenuation methods. China J. Traditional Chin. Med. Pharm. 1-8.

[B36] Feng Y.-Y. Zhou L.-H. Liu N.-N. Sun X.-T. Jia R. Li Q. (2021). Effects of psoralen on invasion and metastasis of human colon cancer cells and β-catenin/TCF4-MMP-9 signaling pathway. China J. Traditional Chin. Med. Pharm. 36 (12), 7033–7037.

[B37] Fu Y. Lee C. H. Chi C. C. (2018). Association of psoriasis with inflammatory bowel disease: a systematic review and meta-analysis. JAMA Dermatol 154 (12), 1417–1423. 10.1001/jamadermatol.2018.3631 30422277 PMC6583370

[B38] Galluzzi L. Pietrocola F. Bravo-San Pedro J. M. Amaravadi R. K. Baehrecke E. H. Cecconi F. (2015). Autophagy in malignant transformation and cancer progression. Embo J. 34 (7), 856–880. 10.15252/embj.201490784 25712477 PMC4388596

[B39] Gao J.-R. Xu S.-Z. Han Y.-Q. Wei L.-B. Song J.-M. (2017). Serum fingerprint of drug-couple Psoralea corylifolia-Myristica fragrants. Chin. Traditional Herb. Drugs 48 (12), 2401–2406.

[B40] Gatenby G. Glyn T. Pearson J. Gearry R. Eglinton T. (2021). The long-term incidence of dysplasia and colorectal cancer in a Crohn's colitis population-based cohort. Colorectal Dis. 23 (9), 2399–2406. 10.1111/codi.15756 34041848

[B41] Ge W. Wang H. Y. Zhao H. M. Liu X. K. Zhong Y. B. Long J. (2020). Effect of sishen pill on memory T cells from experimental colitis induced by dextran sulfate sodium. Front. Pharmacol. 11, 908. 10.3389/fphar.2020.00908 32714185 PMC7343851

[B42] Ge W. Zhou B. G. Zhong Y. B. Liu S. Q. Huang J. Q. Yuan W. Y. (2022). Sishen pill ameliorates dextran sulfate sodium (DSS)-Induced colitis with spleen-kidney yang deficiency syndromes: role of gut microbiota, fecal metabolites, inflammatory dendritic cells, and TLR4/NF-κB pathway. Evid. Based Complement. Altern. Med. 2022, 6132289. 10.1155/2022/6132289 PMC9605852 36310616

[B43] Ghodousi-Dehnavi E. Hosseini R. H. Arjmand M. Nasri S. Zamani Z. (2021). A metabolomic investigation of eugenol on colorectal cancer cell line HT-29 by modifying the expression of APC, p53, and KRAS genes. Evid. Based Complement. Altern. Med. 2021, 1448206. 10.1155/2021/1448206 PMC8616688 34840582

[B44] Guo J. Qiao C. Zhou J. Hu S. Lin X. Shen Y. (2021a). Neobavaisoflavone-mediated T(H)9 cell differentiation ameliorates bowel inflammation. Int. Immunopharmacol. 101 (Pt A), 108191. 10.1016/j.intimp.2021.108191 34601328

[B45] Guo M. Liang D.-Y. Huang R. Pan X. (2023). The application of one test and multiple evaluation method in the content determinationand quality evaluation of 9 components in Sishen pills. J. South-Central Univ. Natl. Sci. Ed. 42 (02), 174–179. 10.20056/j.cnki.ZNMDZK.20230205

[B46] Guo Z. Li P. Wang C. Kang Q. Tu C. Jiang B. (2021b). Five constituents contributed to the Psoraleae fructus-induced hepatotoxicity via mitochondrial dysfunction and apoptosis. Front. Pharmacol. 12, 682823. 10.3389/fphar.2021.682823 34950022 PMC8688997

[B47] He Z. Feng D. Zhang C. Chen Z. Wang H. Hou J. (2024). Recent strategies for evoking immunogenic Pyroptosis in antitumor immunotherapy. J. Control Release 366, 375–394. 10.1016/j.jconrel.2023.12.023 38142962

[B48] Hovhannisyan Z. Treatman J. Littman D. R. Mayer L. (2011). Characterization of interleukin-17-producing regulatory T cells in inflamed intestinal mucosa from patients with inflammatory bowel diseases. Gastroenterology 140 (3), 957–965. 10.1053/j.gastro.2010.12.002 21147109 PMC3049831

[B49] Hsiao S. W. Yen H. H. Chen Y. Y. (2022). Chemoprevention of colitis-associated dysplasia or cancer in inflammatory bowel disease. Gut Liver 16 (6), 840–848. 10.5009/gnl210479 35670121 PMC9668496

[B50] Hu X. Li J. Fu M. Zhao X. Wang W. (2021). The JAK/STAT signaling pathway: from bench to clinic. Signal Transduct. Target Ther. 6 (1), 402. 10.1038/s41392-021-00791-1 34824210 PMC8617206

[B51] Huang J. Chen Z. H. Ren C. M. Wang D. X. Yuan S. X. Wu Q. X. (2015). Antiproliferation effect of evodiamine in human colon cancer cells is associated with IGF-1/HIF-1α downregulation. Oncol. Rep. 34 (6), 3203–3211. 10.3892/or.2015.4309 26503233

[B52] Huang J. Jin J. Kang Z.-P. Liu D.-Y. Cheng S.-M. Zhong Y.-B. (2022). Effect of sisheng pill and its disassembly on expression of Treg cells and PD-1/PD-L1 in mice with colitis. Lishizhen Med. Materia Medica Res. 33 (06), 1284–1287.

[B53] Huang J.-Q. Jiang Q.-Q. Zhong Y.-B. Wang M.-X. Long J. Zhao H.-M. (2021). Regulatory effect of volatile oil from sishenwan on TLR/MyD88 signaling pathway in mice with chronic ulcerative colitis. Chin. J. Exp. Traditional Med. Formulae 27 (23), 19–25. 10.13422/j.cnki.syfjx.20212301

[B54] Huang Y.-L. Huang Z.-P. Wang L.-L. (2019). Simultaneous determination of 8 active components in Sishen pills by flash evaporation-gas chromatography/mass spectrometry. Chin. J. Pharm. Analysis 39 (03), 510–517. 10.16155/j.0254-1793.2019.03.19

[B55] Hung Y. L. Wang S. C. Suzuki K. Fang S. H. Chen C. S. Cheng W. C. (2019). Bavachin attenuates LPS-induced inflammatory response and inhibits the activation of NLRP3 inflammasome in macrophages. Phytomedicine 59, 152785. 10.1016/j.phymed.2018.12.008 31009850

[B56] Hyun S. Y. Le H. T. Min H. Y. Pei H. Lim Y. Song I. (2021). Evodiamine inhibits both stem cell and non-stem-cell populations in human cancer cells by targeting heat shock protein 70. Theranostics 11 (6), 2932–2952. 10.7150/thno.49876 33456581 PMC7806467

[B57] Iacobazzi D. Convertini P. Todisco S. Santarsiero A. Iacobazzi V. Infantino V. (2023). New insights into NF-κB signaling in innate immunity: focus on immunometabolic crosstalks. Biol. (Basel) 12 (6), 776. 10.3390/biology12060776 PMC10294815 37372061

[B58] Ibrahim L. Stanton C. Nutsch K. Nguyen T. Li-Ma C. Ko Y. (2023). Succinylation of a KEAP1 sensor lysine promotes NRF2 activation. Cell Chem. Biol. 30 (10), 1295–1302.e4. 10.1016/j.chembiol.2023.07.014 37619563 PMC10592117

[B59] Ishak N. I. M. Mohamed S. Madzuki I. N. Mustapha N. M. Esa N. M. (2021). Limonin modulated immune and inflammatory responses to suppress colorectal adenocarcinoma in mice model. Naunyn Schmiedeb. Arch. Pharmacol. 394 (9), 1907–1915. 10.1007/s00210-021-02101-6 34009457

[B60] Ismail Abo El-Fadl H. M. Mohamed M. F. A. (2022). Targeting endoplasmic reticulum stress, Nrf-2/HO-1, and NF-κB by myristicin and its role in attenuation of ulcerative colitis in rats. Life Sci. 311 (Pt B), 121187. 10.1016/j.lfs.2022.121187 36403646

[B61] Iwasaki K. Zheng Y. W. Murata S. Ito H. Nakayama K. Kurokawa T. (2016). Anticancer effect of linalool via cancer-specific hydroxyl radical generation in human colon cancer. World J. Gastroenterol. 22 (44), 9765–9774. 10.3748/wjg.v22.i44.9765 27956800 PMC5124981

[B62] Jiang E.-P. Li H. Yu C.-Y. Zhu W. (2015). Influence of schisandrin B in apoptosis and invasion of SW480 cells via p38MAPK signaling pathway. J. Jilin Univ. Ed. 41 (04), 675–679+885. 10.13481/j.1671-587x.20150401

[B63] Jiang Y.-F. Huang Y. Xiao C. Zhou S.-W. Zheng L.-L. You F.-M. (2023). Inhibitory effect and mechanism of sishenwan-containing serum on aerobic glycolysis in human colon cancer cells. Chin. J. Exp. Traditional Med. Formulae 29 (19), 26–33. 10.13422/j.cnki.syfjx.20230130

[B64] Jin J. Liu D.-Y. Huang J. Kang Z.-P. Zhong Y.-B. Long J. (2023). Regulation of sishen pills, ershen pills and wuweizi powder on intestinal microflora lmbalance in mice with colitis. Chin. Archives Traditional Chin. Med. 41 (04), 169–292. 10.13193/j.issn.1673-7717.2023.04.033

[B65] Jin Z. Yan W. Jin H. Ge C. Xu Y. (2016). Differential effect of psoralidin in enhancing apoptosis of colon cancer cells via nuclear factor-κB and B-cell lymphoma-2/B-cell lymphoma-2-associated X protein signaling pathways. Oncol. Lett. 11 (1), 267–272. 10.3892/ol.2015.3861 26870201 PMC4727099

[B66] Kang Z.-P. Jin J. Jiang Q.-Q. Zhao H.-M. Cheng S.-M. Zhong Y.-B. (2022). Effect of Sishen Pills and its split prescriptions on Tfr/Tfh9/Tfh17 cells in colitis mice. China J. Chin. Materia Medica 47 (05), 1300–1306. 10.19540/j.cnki.cjcmm.20211102.401 35343158

[B67] Kilkenny C. Browne W. J. Cuthill I. C. Emerson M. Altman D. G. (2012). Improving bioscience research reporting: the ARRIVE guidelines for reporting animal research. Osteoarthr. Cartil. 20 (4), 256–260. 10.1016/j.joca.2012.02.010 22424462

[B68] Kim B. J. Yang S. K. Kim J. S. Jeen Y. T. Choi H. Han D. S. (2009). Trends of ulcerative colitis-associated colorectal cancer in Korea: a KASID study. J. Gastroenterol. Hepatol. 24 (4), 667–671. 10.1111/j.1440-1746.2008.05730.x 19378391

[B69] Kim H. Yu Y. Choi S. Lee H. Yu J. Lee J. H. (2019). Evodiamine eliminates colon cancer stem cells via suppressing notch and wnt signaling. Molecules 24 (24), 4520. 10.3390/molecules24244520 31835579 PMC6943729

[B70] Kim M. R. Cho S. Y. Lee H. J. Kim J. Y. Nguyen U. T. T. Ha N. M. (2022). Schisandrin C improves leaky gut conditions in intestinal cell monolayer, organoid, and nematode models by increasing tight junction protein expression. Phytomedicine 103, 154209. 10.1016/j.phymed.2022.154209 35689901

[B71] Kong Y.-D. Qi Y. Cui N. Zhang Z.-H. Sun Y.-P. Zeng Y.-N. (2023). Research progress on modern chemical constituents and pharmacological effects of evodia rutaecarpa. Inf. Traditional Chin. Med. 40 (05), 79–83+89. 10.19656/j.cnki.1002-2406.20230513

[B72] Lee A. Chung Y. C. Song K. H. Ryuk J. A. Ha H. Hwang Y. H. (2023). Network pharmacology-based identification of bioavailable anti-inflammatory agents from Psoralea corylifolia L. in an experimental colitis model. J. Ethnopharmacol. 313, 116534. 10.1016/j.jep.2023.116534 37127140

[B73] Lee S. Y. Lee D. Y. Kang J. H. Kim J. H. Jeong J. W. Kim H. W. (2022). Relationship between gut microbiota and colorectal cancer: probiotics as a potential strategy for prevention. Food Res. Int. 156, 111327. 10.1016/j.foodres.2022.111327 35651078

[B74] Li C. Cai G. Song D. Gao R. Teng P. Zhou L. (2019a). Development of EGFR-targeted evodiamine nanoparticles for the treatment of colorectal cancer. Biomater. Sci. 7 (9), 3627–3639. 10.1039/c9bm00613c 31328737

[B75] Li C. Zhang K. Pan G. Ji H. Li C. Wang X. (2021a). Dehydrodiisoeugenol inhibits colorectal cancer growth by endoplasmic reticulum stress-induced autophagic pathways. J. Exp. Clin. Cancer Res. 40 (1), 125. 10.1186/s13046-021-01915-9 33838688 PMC8035743

[B76] Li F. S. Huang J. Cui M. Z. Zeng J. R. Li P. P. Li L. (2020a). BMP9 mediates the anticancer activity of evodiamine through HIF-1α/p53 in human colon cancer cells. Oncol. Rep. 43 (2), 415–426. 10.3892/or.2019.7427 31894286 PMC6967201

[B77] Li J. Lu Y. Wang D. Quan F. Chen X. Sun R. (2019b). Schisandrin B prevents ulcerative colitis and colitis-associated-cancer by activating focal adhesion kinase and influence on gut microbiota in an *in vivo* and *in vitro* model. Eur. J. Pharmacol. 854, 9–21. 10.1016/j.ejphar.2019.03.059 30951716

[B78] Li X. Wu S. Dong G. Chen S. Ma Z. Liu D. (2020b). Natural product evodiamine with borate trigger unit: discovery of potent antitumor agents against colon cancer. ACS Med. Chem. Lett. 11 (4), 439–444. 10.1021/acsmedchemlett.9b00513 32292547 PMC7153022

[B79] Li Y.-G. Deng N. Lin X.-Y. (2018). Meta-analysis of sishen decoction on diarrhea-predominant lrritable bowel syndrome. J. Emerg. Traditional Chin. Med. 27 (02), 215–218.

[B81] Li Z. Qiao L. Yun X. Du F. Xing S. Yang M. (2021b). Increased risk of ischemic heart disease and diabetes in inflammatory bowel disease. Z Gastroenterol. 59 (2), 117–124. 10.1055/a-1283-6966 33233007

[B82] Lim H. S. Kim Y. J. Kim B. Y. Jeong S. J. (2019). Bakuchiol suppresses inflammatory responses via the downregulation of the p38 MAPK/ERK signaling pathway. Int. J. Mol. Sci. 20 (14), 3574. 10.3390/ijms20143574 31336605 PMC6678636

[B83] Liu D.-Y. Xu R. Huang M.-F. Wang X. Zou Y. Yue H.-Y. (2016). Mechanism of sishen wan in regulating balance of T lymphocyte subsets and Treg/Th17 in colitis rats. Chin. J. Exp. Traditional Med. Formulae 22 (03), 107–111. 10.13422/j.cnki.syfjx.2016030107

[B84] Liu L. Zhang L. Cui Z. X. Liu X. Y. Xu W. Yang X. W. (2019). Transformation of psoralen and isopsoralen by human intestinal microbial *in vitro*, and the biological activities of its metabolites. Molecules 24 (22), 4080. 10.3390/molecules24224080 31718071 PMC6891621

[B85] Liu R. Wang Y. Zhu X.-D. Gao Y.-K. Wang H. Zhong X.-T. (2021). Effect of sishenwan on PI3K/Akt/mTOR signal pathway in colonic tissue of rats with ulcerative colitis model of spleen kidney yang deficiency. Chin. J. Exp. Traditional Med. Formulae 27 (04), 16–23. 10.13422/j.cnki.syfjx.20210437

[B86] Liu R.-R. Sun A.-Q. Yu X.-J. He M.-Y. Xie H.-B. Gao P. (2023). Research progress on chemical composition and pharmacological effects of Myristicae Semen and predictive analysis on its quality marker. Chin. Traditional Herb. Drugs 54 (14), 4682–4700.

[B87] Liu S. Zhang S. Lv X. Lu J. Ren C. Zeng Z. (2019). Limonin ameliorates ulcerative colitis by regulating STAT3/miR-214 signaling pathway. Int. Immunopharmacol. 75, 105768. 10.1016/j.intimp.2019.105768 31382166

[B88] Liu S.-P. Ge W. Cheng S.-M. Yuan W.-Y. Zhao H.-M. Liu D.-Y. (2022). Regulation of Sishen Pill on the surface costimulatory molecules of dendritic cells in mice with spleen kidney yang deficiency colitis. Lishizhen Med. Materia Medica Res. 33 (12), 2878–2881.

[B89] Liu W. Liu Y. Wang Z. Yu T. Lu Q. Chen H. (2015). Suppression of MAPK and NF-κ B pathways by schisandrin B contributes to attenuation of DSS-induced mice model of inflammatory bowel disease. Pharmazie 70 (9), 598–603. 26492645

[B90] Liu X. K. Zhao H. M. Wang H. Y. Ge W. Zhong Y. B. Long J. (2020). Regulatory effect of sishen pill on Tfh cells in mice with experimental colitis. Front. Physiol. 11, 589. 10.3389/fphys.2020.00589 32581849 PMC7290041

[B91] Long C.-W. Cao H. (2021a). A meta-analysis on efficacy of modified sishen pill or combined with retention enema in treatment of ulcerative colitis compared with western medicine. J. Pract. Traditional Chin. Intern. Med. 35 (08), 147–148. 10.13729/j.issn.1671-7813.Z20201290

[B92] Long C.-W. Cao H. (2021b). A meta-analysis on efficacy of modified sishen pill or combinedwith retention enema in treatment of ulcerative colitis compared with western medicine. J. Pract. Traditional Chin. Intern. Med. 35 (08), 147–148. 10.13729/j.issn.1671-7813.Z20201290

[B93] Lu C. Xue L. Luo K. Liu Y. Lai J. Yao X. (2023). Colon-accumulated gold nanoclusters alleviate intestinal inflammation and prevent secondary colorectal carcinogenesis via nrf2-dependent macrophage reprogramming. ACS Nano 17 (18), 18421–18432. 10.1021/acsnano.3c06025 37690027

[B94] Lu S. Gong J. Tan Y. Liu D. (2020). Epidemiologic association between inflammatory bowel diseases and type 1 diabetes mellitus: a meta-analysis. J. Gastrointestin Liver Dis. 29 (3), 407–413. 10.15403/jgld-798 32919423

[B95] Luo C. Ai J. Ren E. Li J. Feng C. Li X. (2021). Research progress on evodiamine, a bioactive alkaloid of Evodiae fructus: focus on its anti-cancer activity and bioavailability (Review). Exp. Ther. Med. 22 (5), 1327. 10.3892/etm.2021.10762 34630681 PMC8495584

[B96] Luo W. Lin K. Hua J. Han J. Zhang Q. Chen L. (2022). Schisandrin B attenuates diabetic cardiomyopathy by targeting MyD88 and inhibiting MyD88-dependent inflammation. Adv. Sci. (Weinh) 9 (31), e2202590. 10.1002/advs.202202590 36180407 PMC9631063

[B97] Luo Y. Deng Y. Shen X.-M. Zhang Y. Li J.-C. Li S. (2023). Antidepressant effect of Sishen Wan and its effect on central monoamine nervous system. Chin. J. Pharmacol. Toxicol. 37 (02), 105–111.

[B98] Mayer I. A. Arteaga C. L. (2016). The PI3K/AKT pathway as a target for cancer treatment. Annu. Rev. Med. 67, 11–28. 10.1146/annurev-med-062913-051343 26473415

[B99] Moniri N. H. Farah Q. (2021). Short-chain free-fatty acid G protein-coupled receptors in colon cancer. Biochem. Pharmacol. 186, 114483. 10.1016/j.bcp.2021.114483 33631190

[B100] Morgan X. C. Tickle T. L. Sokol H. Gevers D. Devaney K. L. Ward D. V. (2012). Dysfunction of the intestinal microbiome in inflammatory bowel disease and treatment. Genome Biol. 13 (9), R79. 10.1186/gb-2012-13-9-r79 23013615 PMC3506950

[B101] Mu G.-H. Shi Y. Shen M.-M. Wu L.-T. Zhao Q.-Z. Liu Y.-F. (2018). Overview and thoughts on on the main side effects of fructus Psoraleae. World Chin. Med. 13 (04), 1038–1042.

[B102] Naama M. Telpaz S. Awad A. Ben-Simon S. Harshuk-Shabso S. Modilevsky S. (2023). Autophagy controls mucus secretion from intestinal goblet cells by alleviating ER stress. Cell Host Microbe 31 (3), 433–446.e4. 10.1016/j.chom.2023.01.006 36738733 PMC10016318

[B103] Ng S. C. Shi H. Y. Hamidi N. Underwood F. E. Tang W. Benchimol E. I. (2017). Worldwide incidence and prevalence of inflammatory bowel disease in the 21st century: a systematic review of population-based studies. Lancet 390 (10114), 2769–2778. 10.1016/s0140-6736(17)32448-0 29050646

[B104] Park M. H. Kim J. H. Chung Y. H. Lee S. H. (2016). Bakuchiol sensitizes cancer cells to TRAIL through ROS- and JNK-mediated upregulation of death receptors and downregulation of survival proteins. Biochem. Biophys. Res. Commun. 473 (2), 586–592. 10.1016/j.bbrc.2016.03.127 27033605

[B105] Piovani D. Hassan C. Repici A. Rimassa L. Carlo-Stella C. Nikolopoulos G. K. (2022). Risk of cancer in inflammatory bowel diseases: umbrella review and reanalysis of meta-analyses. Gastroenterology 163 (3), 671–684. 10.1053/j.gastro.2022.05.038 35643170

[B106] Piras A. Rosa A. Marongiu B. Atzeri A. Dessì M. A. Falconieri D. (2012). Extraction and separation of volatile and fixed oils from seeds of Myristica fragrans by supercritical CO₂: chemical composition and cytotoxic activity on Caco-2 cancer cells. J. Food Sci. 77 (4), C448–C453. 10.1111/j.1750-3841.2012.02618.x 22429024

[B107] Pt H. J. Sally G. (2024). Cochrane Handbook for systematic reviews of interventions:cochrane book series. John Wiley & Sons, Ltd.

[B108] Pu Z. Zhang W. Wang M. Xu M. Xie H. Zhao J. (2021). Schisandrin B attenuates colitis-associated colorectal cancer through SIRT1 linked SMURF2 signaling. Am. J. Chin. Med. 49 (7), 1773–1789. 10.1142/s0192415x21500841 34632965

[B109] Qi Y.-T. Zhang F.-R. Miao Y.-L. (2021). Pyropotosis and inflammatory bowel disease. Chin. J. Inflamm. Bowel Dis. 05 (1), 92–95. 10.3760/cma.j.cn101480-20191022-00127

[B110] Rath T. Atreya R. Bodenschatz J. Uter W. Geppert C. E. Vitali F. (2023). Intestinal barrier healing is superior to endoscopic and histologic remission for predicting major adverse outcomes in inflammatory bowel disease: the prospective ERIca trial. Gastroenterology 164 (2), 241–255. 10.1053/j.gastro.2022.10.014 36279923

[B111] Ren Y. Song X. Tan L. Guo C. Wang M. Liu H. (2020). A review of the pharmacological properties of psoralen. Front. Pharmacol. 11, 571535. 10.3389/fphar.2020.571535 33013413 PMC7500444

[B112] Ronkina N. Gaestel M. (2022). MAPK-activated protein kinases: servant or partner? Annu. Rev. Biochem. 91, 505–540. 10.1146/annurev-biochem-081720-114505 35303787

[B113] Saito T. Nishikawa H. Wada H. Nagano Y. Sugiyama D. Atarashi K. (2016). Two FOXP3(+)CD4(+) T cell subpopulations distinctly control the prognosis of colorectal cancers. Nat. Med. 22 (6), 679–684. 10.1038/nm.4086 27111280

[B114] Salas A. Hernandez-Rocha C. Duijvestein M. Faubion W. McGovern D. Vermeire S. (2020). JAK-STAT pathway targeting for the treatment of inflammatory bowel disease. Nat. Rev. Gastroenterol. Hepatol. 17 (6), 323–337. 10.1038/s41575-020-0273-0 32203403

[B115] Shah S. C. Itzkowitz S. H. (2022). Colorectal cancer in inflammatory bowel disease: mechanisms and management. Gastroenterology 162 (3), 715–730.e3. 10.1053/j.gastro.2021.10.035 34757143 PMC9003896

[B116] Shalapour S. Karin M. (2020). Cruel to Be kind: epithelial, microbial, and immune cell interactions in gastrointestinal cancers. Annu. Rev. Immunol. 38, 649–671. 10.1146/annurev-immunol-082019-081656 32040356 PMC8936002

[B117] Shao B. Tang J. Ji H. Liu H. Liu Y. Zhu D. (2010). Enhanced oral bioavailability of Wurenchun (Fructus Schisandrae Chinensis extracts) by self-emulsifying drug delivery systems. Drug Dev. Ind. Pharm. 36 (11), 1356–1363. 10.3109/03639045.2010.480975 20849350

[B118] Sharifi-Rad J. Kamiloglu S. Yeskaliyeva B. Beyatli A. Alfred M. A. Salehi B. (2020). Pharmacological activities of psoralidin: a comprehensive review of the molecular mechanisms of action. Front. Pharmacol. 11, 571459. 10.3389/fphar.2020.571459 33192514 PMC7643726

[B119] Sheikh B. Y. Sarker M. M. R. Kamarudin M. N. A. Mohan G. (2017). Antiproliferative and apoptosis inducing effects of citral via p53 and ROS-induced mitochondrial-mediated apoptosis in human colorectal HCT116 and HT29 cell lines. Biomed. Pharmacother. 96, 834–846. 10.1016/j.biopha.2017.10.038 29078261

[B120] Shen P. Zhang Z. Zhu K. Cao H. Liu J. Lu X. (2019). Evodiamine prevents dextran sulfate sodium-induced murine experimental colitis via the regulation of NF-κB and NLRP3 inflammasome. Biomed. Pharmacother. 110, 786–795. 10.1016/j.biopha.2018.12.033 30554117

[B121] Sohn J. H. Han K. L. Kim J. H. Rukayadi Y. Hwang J. K. (2008). Protective Effects of macelignan on cisplatin-induced hepatotoxicity is associated with JNK activation. Biol. Pharm. Bull. 31 (2), 273–277. 10.1248/bpb.31.273 18239286

[B122] Song C. Chen J. Li X. Yang R. Cao X. Zhou L. (2021). Limonin ameliorates dextran sulfate sodium-induced chronic colitis in mice by inhibiting PERK-ATF4-CHOP pathway of ER stress and NF-κB signaling. Int. Immunopharmacol. 90, 107161. 10.1016/j.intimp.2020.107161 33168409

[B123] Sun C. Zhao L. Wang X. Hou Y. Guo X. Lu J. J. (2022). Psoralidin, a natural compound from Psoralea corylifolia, induces oxidative damage mediated apoptosis in colon cancer cells. J. Biochem. Mol. Toxicol. 36 (7), e23051. 10.1002/jbt.23051 35315184

[B124] Sun H.-H. Yang J. Wang D.-J. (2021). Clinical study of Lizhong decoction and Sishen pill modified and subtractedcombined with FOLFIRl chemotherapy on elderly patients with advanced colon cancer with spleen and kidney Yang deficiency syndrome. Chin. J. Integr. Traditional West. Med. Dig. 29 (04), 276–279.

[B125] Sun M. Wu W. Liu Z. Cong Y. (2017). Microbiota metabolite short chain fatty acids, GPCR, and inflammatory bowel diseases. J. Gastroenterol. 52 (1), 1–8. 10.1007/s00535-016-1242-9 27448578 PMC5215992

[B126] Tan Y.-B. Jiang L.-J. Liao Z.-J. Zhou Q. (2023). Clinical effect of Sishen pill combined with Lizhong decoction in the treatment of patients with spleen-kidney Yang deficiency syndrome after colorectal cancer surgery based on intestinal flora. China Med. 18 (07), 1054–1058.

[B127] Taniguchi K. Karin M. (2018). NF-κB, inflammation, immunity and cancer: coming of age. Nat. Rev. Immunol. 18 (5), 309–324. 10.1038/nri.2017.142 29379212

[B128] Taylor C. T. Colgan S. P. (2007). Hypoxia and gastrointestinal disease. J. Mol. Med. Berl. 85 (12), 1295–1300. 10.1007/s00109-007-0277-z 18026919

[B129] Tekeli I. O. Ateşşahin A. Sakin F. Aslan A. Çeribaşı S. Yipel M. (2018). Protective effects of conventional and colon-targeted lycopene and linalool on ulcerative colitis induced by acetic acid in rats. Inflammopharmacology 27, 313–322. 10.1007/s10787-018-0485-x 29736689

[B130] van der Post S. Jabbar K. S. Birchenough G. Arike L. Akhtar N. Sjovall H. (2019). Structural weakening of the colonic mucus barrier is an early event in ulcerative colitis pathogenesis. Gut 68 (12), 2142–2151. 10.1136/gutjnl-2018-317571 30914450 PMC6872445

[B131] Wan M. L. Y. Turner P. C. Co V. A. Wang M. F. Amiri K. M. A. El-Nezami H. (2019). Schisandrin A protects intestinal epithelial cells from deoxynivalenol-induced cytotoxicity, oxidative damage and inflammation. Sci. Rep. 9 (1), 19173. 10.1038/s41598-019-55821-4 31844123 PMC6915730

[B132] Wang A.-H. He L.-J. Zhu X.-D. (2019a). Effect of sishenwan on toll-like receptor 4 and lRAK-M expression in colonic tissue of rats with ulcerative colitis of spleen-kidney yang deficiency type. Chin. J. Exp. Traditional Med. Formulae 25 (14), 70–76. 10.13422/j.cnki.syfjx.20191439

[B133] Wang D. Ge S. Chen Z. Song Y. (2019b). Evodiamine exerts anticancer effects via induction of apoptosis and autophagy and suppresses the migration and invasion of human colon cancer cells. J. buon 24 (5), 1824–1829. 31786843

[B134] Wang G. Yu Y. Wang Y. Z. Wang J. J. Guan R. Sun Y. (2019c). Role of SCFAs in gut microbiome and glycolysis for colorectal cancer therapy. J. Cell Physiol. 234 (10), 17023–17049. 10.1002/jcp.28436 30888065

[B135] Wang H. Y. Zhao H. M. Wang Y. Liu Y. Lu X. Y. Liu X. K. (2019d). Sishen Wan® ameliorated trinitrobenzene-sulfonic-acid-induced chronic colitis via NEMO/NLK signaling pathway. Front. Pharmacol. 10, 170. 10.3389/fphar.2019.00170 30894816 PMC6414459

[B136] Wang L. Fang K. Cheng J. Li Y. Huang Y. Chen S. (2020a). Scaffold hopping of natural product evodiamine: discovery of a novel antitumor scaffold with excellent potency against colon cancer. J. Med. Chem. 63 (2), 696–713. 10.1021/acs.jmedchem.9b01626 31880942

[B137] Wang L. He C. (2022). Nrf2-mediated anti-inflammatory polarization of macrophages as therapeutic targets for osteoarthritis. Front. Immunol. 13, 967193. 10.3389/fimmu.2022.967193 36032081 PMC9411667

[B138] Wang L. Tang L. Feng Y. Zhao S. Han M. Zhang C. (2020b). A purified membrane protein from Akkermansia muciniphila or the pasteurised bacterium blunts colitis associated tumourigenesis by modulation of CD8(+) T cells in mice. Gut 69 (11), 1988–1997. 10.1136/gutjnl-2019-320105 32169907 PMC7569398

[B139] Wang L.-H. Ma D.-C. Sun L.-X. (2015). The origin and new exploration of SiShen pills. West. J. Traditional Chin. Med. 28 (03), 47–49.

[B140] Wang M. Huang X. Kang Z. Huang J. Wei S. Zhao H. (2022a). Mechanism of sishen-pill-regulated special memory T and mTfh cell via involving JAK/STAT5 pathway in colitis mice. Evid. Based Complement. Altern. Med. 2022, 6446674. 10.1155/2022/6446674 PMC8979676 35388299

[B141] Wang M. Tian B. Shen J. Xu S. Liu C. Guan L. (2023a). Bavachin induces apoptosis in colorectal cancer cells through Gadd45a via the MAPK signaling pathway. Chin. J. Nat. Med. 21 (1), 36–46. 10.1016/s1875-5364(23)60383-8 36641231

[B142] Wang M. Zhou B. Cong W. Zhang M. Li Z. Li Y. (2021). Amelioration of AOM/DSS-Induced murine colitis-associated cancer by evodiamine intervention is primarily associated with gut microbiota-metabolism-inflammatory signaling Axis. Front. Pharmacol. 12, 797605. 10.3389/fphar.2021.797605 35002731 PMC8740177

[B143] Wang M. X. Lin L. Chen Y. D. Zhong Y. P. Lin Y. X. Li P. (2020c). Evodiamine has therapeutic efficacy in ulcerative colitis by increasing Lactobacillus acidophilus levels and acetate production. Pharmacol. Res. 159, 104978. 10.1016/j.phrs.2020.104978 32485282

[B144] Wang S.-J. Zhang Y. Liang H.-Y. Zhang M.-B. Xu Z.-L. (2023b). Influence of deoxyschizandrin on NF KB/COX-2 signal pathway in colon of mice with inflammatory bowel disease. West. J. Traditional Chin. Med. 36 (09), 35–39.

[B145] Wang T. Shen Y. Luyten S. Yang Y. Jiang X. (2020d). Tissue-resident memory CD8(+) T cells in cancer immunology and immunotherapy. Pharmacol. Res. 159, 104876. 10.1016/j.phrs.2020.104876 32422340

[B146] Wang W. Wang S. K. Wang Q. Zhang Z. Li B. Zhou Z. D. (2023c). Diclofenac and eugenol hybrid with enhanced anti-inflammatory activity through activating HO-1 and inhibiting NF-κB pathway *in vitro* and *in vivo* . Eur. J. Med. Chem. 259, 115669. 10.1016/j.ejmech.2023.115669 37517204

[B147] Wang X. Shen C. Wang X. Tang J. Wu Z. Huang Y. (2023d). Schisandrin protects against ulcerative colitis by inhibiting the SGK1/NLRP3 signaling pathway and reshaping gut microbiota in mice. Chin. Med. 18 (1), 112. 10.1186/s13020-023-00815-8 37674245 PMC10481484

[B148] Wang Y. Zhang T. He X. (2023e). Advances in the role of microRNAs associated with the PI3K/AKT signaling pathway in lung cancer. Front. Oncol. 13, 1279822. 10.3389/fonc.2023.1279822 38169723 PMC10758458

[B149] Wang Y. Zhu X. Liang Y. Li X. Wang Y. Li J. (2022b). Sishen wan treats ulcerative colitis in rats by regulating gut microbiota and restoring the Treg/Th17 balance. Evid. Based Complement. Altern. Med. 2022, 1432816. 10.1155/2022/1432816 PMC9822768 36619196

[B150] Wang Y. F. Liu Y. N. Xiong W. Yan D. M. Zhu Y. Gao X. M. (2014). A UPLC-MS/MS method for *in vivo* and *in vitro* pharmacokinetic studies of psoralenoside, isopsoralenoside, psoralen and isopsoralen from Psoralea corylifolia extract. J. Ethnopharmacol. 151 (1), 609–617. 10.1016/j.jep.2013.11.013 24315982

[B151] Wang Z. J. Chen L. H. Xu J. Xu Q. X. Xu W. Yang X. W. (2023f). Corylin ameliorates chronic ulcerative colitis via regulating the gut-brain axis and promoting 5-hydroxytryptophan production in the colon. Phytomedicine 110, 154651. 10.1016/j.phymed.2023.154651 36634380

[B152] Wei S.-T. Liu Y.-Q. Huang J. Sheng Y.-H. Tang L.-M. (2020). Research progress of chemical component,Medicinal efficacy and liver toxicity of fructus evodiae. World Chin. Med. 15 (23), 3580–3585+3592.

[B153] Wei W. Hou J.-Z. Zhu S.-J. Sheng X.-Y. Guo H.-R. (2021). Study on quality control of multi-components in sishen pills based on fingerprints. Chin. J. Inf. Traditional Chin. Med. 28 (10).

[B154] Wu W. Liu Z.-J. (2022). Mechanisms of the carcinogenesis of inflammatory bowel disease and current status of animal models researches. Chin. J. Inflamm. Bowel Dis. 06 (4), 281–286. 10.3760/cma.j.cn101480-20220913-00146

[B155] Xing N.-N. Qu H.-D. Ren W.-C. Ma W. (2021). Main chemical constituents and modern pharmacological action of Schisandrae chinensis fructus:A review. Chin. J. Exp. Traditional Med. Formulae 27 (15), 210–218. 10.13422/j.cnki.syfjx.20211407

[B156] Xu B. Xiao L.-B. (2023). Research Progress on hepatotoxicity and attenuation of Psoralea corylifolia. Lishizhen Med. Materia Medica Res. 34 (01), 159–161.

[B157] Xu J. Zhang Q. Wang H. Zhang Y. Cheng P.-Y. Gao F. (2022a). Clinical observation on the efficacy and adverse reaction of modified sishen pill in combination with abemaciclib and endocrine therapy in patients with HR+/HER2-Advanced breast cancer. Chin. J. Ration. Drug Use 19 (12), 38–43.

[B158] Xu Z.-L. Xu H. Chen X. Li R.-N. Tao X.-J. Li X.-L. (2022b). Meta-analysis of Sishen-pill alone or in combination in the treatment of inflammatory bowel disease. China Mod. Dr. 60 (29), 72–75+93.

[B159] Xue M. Shi L. Wang W. Chen S. Wang L. (2018). An overview of molecular profiles in ulcerative colitis-related cancer. Inflamm. Bowel Dis. 24 (9), 1883–1894. 10.1093/ibd/izy221 29945208

[B160] Yamazaki T. Bravo-San Pedro J. M. Galluzzi L. Kroemer G. Pietrocola F. (2021). Autophagy in the cancer-immunity dialogue. Adv. Drug Deliv. Rev. 169, 40–50. 10.1016/j.addr.2020.12.003 33301821

[B161] Yang C.-Q. Lian W.-Y. Wang Y.-G. Gao Y. (2021). Research progress in pharmacology and toxicology of evodiamine. China J. Chin. Materia Medica 46 (20), 5218–5225. 10.19540/j.cnki.cjcmm.20210518.602 34738422

[B163] Yang T. Li S. Deng S.-Q. (2023a). Mechanism of isobavachalcone in treatment of ulcerative colitis. J. Shanghai Univ. Sci. Ed. 29 (02), 253–263.

[B164] Yang W. Gong X. Wang X. Huang C. (2019). A mediator of phosphorylated Smad2/3, evodiamine, in the reversion of TAF-induced EMT in normal colonic epithelial cells. Invest New Drugs 37 (5), 865–875. 10.1007/s10637-018-0702-x 30488243

[B165] Yang X. N. Liu X. M. Fang J. H. Zhu X. Yang X. W. Xiao X. R. (2018). PPARα mediates the hepatoprotective effects of nutmeg. J. Proteome Res. 17 (5), 1887–1897. 10.1021/acs.jproteome.7b00901 29664296 PMC6628927

[B166] Yang Y. Liu Z. Lyu H. Guo X. Jiang H. Liu L. (2023b). Traditional Chinese medicine-induced treatment in colitis-associated colorectal cancer. Chin. Med. J. Engl. 136 (10), 1249–1250. 10.1097/cm9.0000000000002667 37036895 PMC10278721

[B167] Yimam M. Jiao P. Hong M. Jia Q. (2016). Hepatoprotective activity of an herbal composition, MAP, a standardized blend comprising Myristica fragrans, Astragalus membranaceus, and poria cocos. J. Med. Food 19 (10), 952–960. 10.1089/jmf.2016.0048 27564381

[B168] Yong H. Y. Koh M. S. Moon A. (2009). The p38 MAPK inhibitors for the treatment of inflammatory diseases and cancer. Expert Opin. Investig. Drugs 18 (12), 1893–1905. 10.1517/13543780903321490 19852565

[B169] Yu S. Qian H. (2021). Deoxyschizandrin treats mice with ulcerative colitis possibly via the TLR4/NF-κB signaling pathway. Am. J. Transl. Res. 13 (4), 3856–3863. 34017577 PMC8129281

[B170] Yu W.-L. Wang Y. Cheng J. Niu L.-H. Chen G.-Y. An F.-L. (2024). Effect of sishen pill and disassembling prescription on experimental ulcerative colitis in rats. Pharmacol. Clin. Chin. Materia Medica, 1–17. 10.13412/j.cnki.zyyl.20231020.001

[B171] Zaka A. Mridha N. Subhaharan D. Jones M. Niranjan S. Mohsen W. (2023). Inflammatory bowel disease patients have an increased risk of acute coronary syndrome: a systematic review and meta-analysis. Open Heart 10 (2), e002483. 10.1136/openhrt-2023-002483 37940332 PMC10632902

[B172] Zangara M. T. Darwish L. Coombes B. K. (2023). Characterizing the pathogenic potential of Crohn's disease-associated adherent-invasive *Escherichia coli* . EcoSal Plus 11, eesp00182022. 10.1128/ecosalplus.esp-0018-2022 37220071 PMC10729932

[B173] Zhang C. Fan X. Xu X. Yang X. Wang X. Liang H. P. (2010). Evodiamine induces caspase-dependent apoptosis and S phase arrest in human colon lovo cells. Anticancer Drugs 21 (8), 766–776. 10.1097/CAD.0b013e32833d26a9 20647931

[B174] Zhang G. Yu Y. Huang H.-L. Zhang S.-C. (2019). Mechanism of sishenwan in treatment of ulcerative colitis based on network pharmacology and bioinformatics. Chin. J. Exp. Traditional Med. Formulae 25 (24), 142–149. 10.13422/j.cnki.syfjx.20192438

[B175] Zhang W. Wang W. Shen C. Wang X. Pu Z. Yin Q. (2021a). Network pharmacology for systematic understanding of Schisandrin B reduces the epithelial cells injury of colitis through regulating pyroptosis by AMPK/Nrf2/NLRP3 inflammasome. Aging (Albany NY) 13 (19), 23193–23209. 10.18632/aging.203611 34628369 PMC8544312

[B176] Zhang W. F. Yang Y. Su X. Xu D. Y. Yan Y. L. Gao Q. (2016). Deoxyschizandrin suppresses dss-induced ulcerative colitis in mice. Saudi J. Gastroenterol. 22 (6), 448–455. 10.4103/1319-3767.195552 27976641 PMC5184746

[B177] Zhang X.-X. Jin J.-W. Liu C.-H. Zhou M. He Y.-X. Wang F. (2021b). Effect of Nrf2/HO-1 signaling pathway in intestinal protection by Sishen Pills against ulcerative colitis in mice. China J. Chin. Materia Medica 46 (16), 4187–4192. 10.19540/j.cnki.cjcmm.20210524.402 34467731

[B178] Zhang X.-X. Li X.-N. Hu S. Chang R.-M. (2018). Simultaneous determination of nine bioactive components in Sishen Pills by HPLC-ESI-MS/MS. Chin. Traditional Herb. Drugs 49 (09), 2070–2075.

[B179] Zhang X.-Y. Zhao H.-M. Liu Y. Liu X.-K. Liu F.-C. Chen F. (2020a). Regulation of schisandrin A on oxidative stress and ulcerative colitis in rats. Chin. Archives Traditional Chin. Med. 38 (02), 166–169+283. 10.13193/j.issn.1673-7717.2020.02.041

[B180] Zhang X. Y. Zhao H. M. Liu Y. Lu X. Y. Li Y. Z. Pan Q. H. (2021c). Sishen pill maintained colonic mucosal barrier integrity to treat ulcerative colitis via Rho/ROCK signaling pathway. Evid. Based Complement. Altern. Med. 2021, 5536679. 10.1155/2021/5536679 PMC8677397 34925530

[B181] Zhang Y. Dai H.-B. Zhu P.-F. (2021d). Observation on the curative effect of shenling baizhu powder and sishen Pill Combined with chemotherapy for radical resection of colorectal cancer. Chin. J. Surg. Integr. Traditional West. Med. 27 (04), 592–596.

[B182] Zhang Y. Yan T. Sun D. Xie C. Wang T. Liu X. (2020b). Rutaecarpine inhibits KEAP1-NRF2 interaction to activate NRF2 and ameliorate dextran sulfate sodium-induced colitis. Free Radic. Biol. Med. 148, 33–41. 10.1016/j.freeradbiomed.2019.12.012 31874248 PMC7376370

[B183] Zhang Y. Zhang H. Zhang K. Li Z. Guo T. Wu T. (2020c). Co-hybridized composite nanovesicles for enhanced transdermal eugenol and cinnamaldehyde delivery and their potential efficacy in ulcerative colitis. Nanomedicine 28, 102212. 10.1016/j.nano.2020.102212 32334099

[B184] Zhang Y. Zhang Y. Zhao Y. Wu W. Meng W. Zhou Y. (2022). Protection against ulcerative colitis and colorectal cancer by evodiamine via anti-inflammatory effects. Mol. Med. Rep. 25 (5), 188. 10.3892/mmr.2022.12704 35362542 PMC8985202

[B185] Zhao H. Ming T. Tang S. Ren S. Yang H. Liu M. (2022). Wnt signaling in colorectal cancer: pathogenic role and therapeutic target. Mol. Cancer 21 (1), 144. 10.1186/s12943-022-01616-7 35836256 PMC9281132

[B186] Zhao H. M. Huang X. Y. Zhou F. Tong W. T. Wan P. T. Huang M. F. (2013). Si shen wan inhibits mRNA expression of apoptosis-related molecules in p38 MAPK signal pathway in mice with colitis. Evid. Based Complement. Altern. Med. 2013, 432097. 10.1155/2013/432097 PMC3816044 24223057

[B187] Zhao H. M. Liu Y. Huang X. Y. Liu X. K. Chen F. Zhang X. Y. (2019). Pharmacological mechanism of Sishen Wan® attenuated experimental chronic colitis by inhibiting wnt/β-catenin pathway. J. Ethnopharmacol. 240, 111936. 10.1016/j.jep.2019.111936 31078692

[B188] Zhao Q. Duck L. W. Huang F. Alexander K. L. Maynard C. L. Mannon P. J. (2020). CD4(+) T cell activation and concomitant mTOR metabolic inhibition can ablate microbiota-specific memory cells and prevent colitis. Sci. Immunol. 5 (54), eabc6373. 10.1126/sciimmunol.abc6373 33310866

[B189] Zhaohua Z. Rong L. Nana D. U. Xiangdong Z. (2022). Efficacy of Sishen Wan on dinitrobenzene sulfonic acid-induced ulcerative colitis and its effect on toll-like receptor 2/interleukin-1 receptor-associated kinase-4/nuclear factor-κB signal pathway. J. Tradit. Chin. Med. 42 (4), 565–575. 10.19852/j.cnki.jtcm.20220608.001 35848973 PMC9924653

[B190] Zhou Z.-X. (2020). The screening of active ingredients and underlying mechanism in Psoraleae Fructus treatment of ulcerative colitis. doctor. doctor’s thesis. Liaoning: Shenyang Pharmaceutical University.

[B191] Zhu L. Q. Zhang L. Zhang J. Chang G. L. Liu G. Yu D. D. (2021). Evodiamine inhibits high-fat diet-induced colitis-associated cancer in mice through regulating the gut microbiota. J. Integr. Med. 19 (1), 56–65. 10.1016/j.joim.2020.11.001 33277208

